# Predicting Bioconcentration Factors and Concentrations of Per- and Polyfluoroalkyl Substances in Plants with Machine Learning

**DOI:** 10.1021/acs.est.6c10458

**Published:** 2026-09-01

**Authors:** Rui Li, Jiyoon Yi, Hui Li, Wei Zhang

**Affiliations:** † Department of Plant, Soil and Microbial Sciences, 3078 Michigan State University, East Lansing, Michigan 48824, United States; ‡ Department of Biosystems and Agricultural Engineering, Michigan State University, East Lansing, Michigan 48824, United States; § Environmental Science and Policy Program, Michigan State University, East Lansing, Michigan 48824, United States

**Keywords:** Per- and polyfluoroalkyl substances, PFAS, bioaccumulation, supervised machine learning, permutation feature importance, SHAP analysis

## Abstract

Bioconcentration factors (BCF) and concentrations of per- and polyfluoroalkyl substances (PFAS) in crops were predicted by four machine learning models using a newly compiled data set composed of 11 crops, 11 plant tissue compartments, and 21 PFAS, as well as diverse soil properties and cultivation conditions. The eXtreme Gradient Boosting (XGBoost) achieved the best performance, with a coefficient of determination (*R* ^2^) of 0.951 for Log BCF and 0.943 for Log C. Feature importance analysis showed that soil pH and PFAS carbon chain length are key predictors of BCF. Soil PFAS concentration is the primary input feature for PFAS concentrations in plants and shorter growth-cycle plants accumulated higher PFAS concentrations. Root and leaf tissues were distinguished as the primary compartments of PFAS accumulation, exhibiting the highest BCF and concentration levels. Interestingly, although PFAS accumulation in plants generally shows a negative correlation with carbon chain length, root accumulation has a relatively weaker correlation with high levels of accumulation for both short-chain and long-chain PFAS. In contrast, above-ground tissues (particularly leaves) preferentially accumulate short-chain PFAS. These findings could provide valuable insights into the development of management and mitigation strategies to minimize PFAS accumulation in food crops and subsequently human exposure through the food chain.

## Graphical abstract

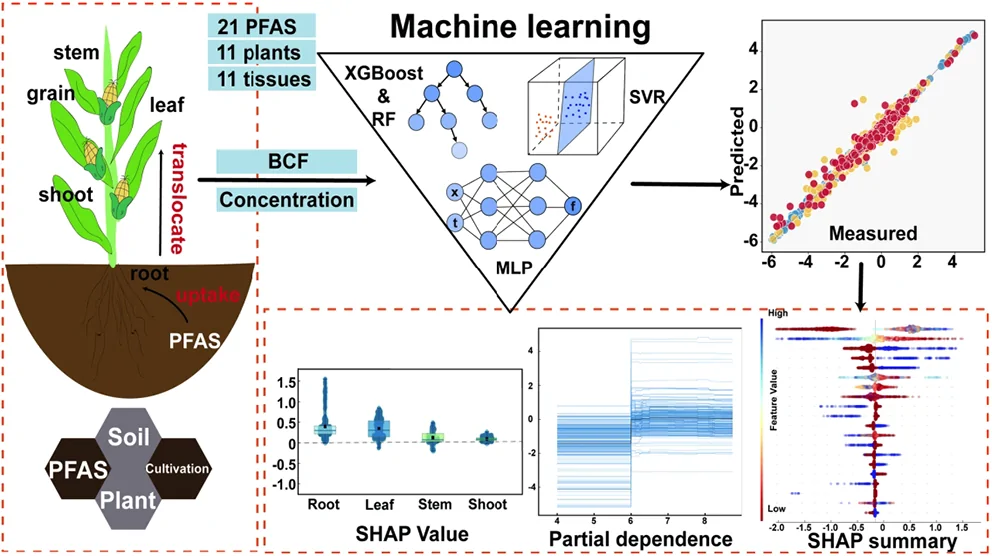

## 1 Introduction

Per- and polyfluoroalkyl substances (PFAS) are widely used in industries and consumer products due to their unique interfacial activity and remarkable chemical stability.^1^ PFAS are globally distributed in various environments and are of significant environmental and health concerns due to their persistence, bioaccumulation, and toxicity.^2^ Many PFAS have been shown to accumulate in protein-rich organs, tissues, and body fluids, such as liver, kidney, human serum, and breast milk,^3^ with half-lives ranging from several years to decades.^4^ Certain PFAS such as perfluorooctanoic acid (PFOA) and perfluorooctanesulfonate (PFOS) exhibit hepatotoxicity, neurotoxicity, reproductive and developmental toxicity, endocrine disruption, and immunotoxicity.^5^ In fact, PFOA has recently been classified as carcinogenic to humans (Group 1) and PFOS possibly carcinogenic to humans (Group 2B) by the International Agency for Research on Cancer (IARC).^6^ These adverse human risks have raised significant concerns over PFAS exposure through food and drinking water.^7^ In 2020, the European Food Safety Authority (EFSA) established a tolerable weekly intake (TWI) of 4.4 ng/kg body weight per week for the sum of PFOA, PFNA, PFHxS and PFOS, increasingly recognizing dietary intake as a major exposure pathway to PFAS for humans.^8^ Food crops including vegetables and field crops have been shown to accumulate PFAS to the ng/g−μg/g levels.^9^ For example, field experiments have shown significant accumulation of perfluorobutanoic acid (PFBA) in soybeans up to 1078 ng/g, wheat leaf 1103 ng/g, wheat grains 342 ng/g, and corn leaf 1449 ng/g, respectively.^10^ Leafy vegetables such as lettuce tend to accumulate higher levels of PFAS than cereals, with leaves containing up to 2365 ng/g of PFBA and 1038 ng/g of PFOA.^10^ Similarly, radish shoots have been found to accumulate PFBA and PFOA at concentrations of 1168 and 1880 ng/g, respectively.^10^ Thus, it is important to study the mechanisms and key factors responsible for the uptake and accumulation of PFAS in plants.

Fundamentally, PFAS sorbed in soils can be continuously desorbed into soil pore water, thereby becoming bioavailable for plant root uptake.^11^ Many soil pot and field studies have been conducted to explore influencing factors for PFAS uptake by plants. Overall, the uptake and accumulation of PFAS in plants is influenced by physicochemical properties of PFAS, soil properties, and plant-specific traits.^12^ Molecular structure and carbon chain length of PFAS highly correlate with their uptake by plant roots.^13^ PFAS with longer carbon chains have larger molecular size, as well as higher Log *K*_OW_ values (typically >4) and hydrophobicity, making them more strongly sorbed by soils or retained in roots once taken up by plants.^14^ Soil properties, including cation exchange capacity (CEC), pH, temperature, salinity, organic carbon content, and clay content, also significantly affect the bioavailability and plant uptake of PFAS.^15,16^ Plant species, cultivars, and tissue compartments are the main biotic factors that affect the behavior of PFAS within plants.^17,9,18^ Additionally, some studies have investigated the effects of root traits (e.g., protein and lipid content) and transpiration on the accumulation of PFAS in plants.^19,20,17^

Nonetheless, these traditional experimental approaches are often resource-intensive and time-consuming, resulting in a limited number of PFAS and plants in any given study. Thus, there is a lack of systematic evaluation to fully capture complex interactions among PFAS, soils, plants, and cultivation conditions in experimental studies. Machine learning can be a powerful tool to explore complex interactions, reveal key predictors, and generate reliable predictions,^21^ which allows for more in-depth analysis of existing data sets on plant uptake of PFAS. Machine learning has been used to predict environmental occurrence in soils, groundwater, and organisms (e.g., fish tissues),^22–24^ bioactivity,^25,26^ solid–liquid distribution,^27^ toxicity,^28^ defluorination potential,^29^ exposure and health risks,^30^ as well as plant uptake and accumulation of PFAS.^31,32^ Previous studies used various machine learning models to achieve strong predictive performance and identify the most influential features or factors for plant uptake of PFAS. Recent studies, for example, developed machine learning models to predict root concentration factors using similar feature sets, only focusing on bioconcentration factors (BCF).^31–33^ However, a few aspects remain to be improved. First, the previous machine learning models exclusively predict BCF or bioaccumulation factors (BAF), which were originally introduced for evaluating phytoremediation potential^34^ and subsequently adopted to quantify bioaccumulation in edible crops. These parameters assume a quasi-equilibrium partitioning process and are influenced by plant species, plant growth time, lipid content, soil properties (including contaminant concentrations), and contaminant properties.^35–37^ Interestingly, conflicting trends were reported on the changes of BCFs with increasing soil PFAS levels,^18,31^ leading to unreliable estimation of plant concentrations at realistic soil PFAS levels. Furthermore, no existing model provides tissue-specific concentration estimates needed for dietary risk assessment. We intended to bridge this gap by applying machine learning models that directly predict absolute PFAS concentrations in edible plant tissues across environmentally relevant soil PFAS concentrations. By modeling BCF as a normalized indicator of accumulation potential and absolute PFAS concentrations in plant tissues, our models allow for both comparative analysis of accumulation drivers and direct estimation of tissue PFAS concentrations for dietary exposure assessment. Finally, the previous machine learning models have not thoroughly investigated the differences in PFAS uptake and accumulation among various plant tissues, particularly between roots and aboveground parts,^38^ which is crucial to elucidation of the PFAS uptake mechanisms, robust risk assessment, and development of mitigation strategies.

To systematically study the plant uptake of PFAS, we applied and evaluated four supervised machine learning regression models, specifically Random Forest (RF), eXtreme Gradient Boosting (XGBoost), Multilayer Perceptron (MLP), and Support Vector Regression (SVR), using our recently compiled data set. This comprehensive data set includes a wide range of variables, such as 11 crop types, 11 plant tissue compartments, and 21 PFAS compounds, as well as diverse soil properties, cultivation conditions, and structural features of PFAS. By doing so we were able to (1) train machine learning models capable of achieving high predictive performance for BCF and PFAS concentrations in various plant tissues; (2) identify the key features to BCF and PFAS concentrations in plants; and (3) investigate the differences in PFAS uptake and accumulation between plant roots and above-ground parts and the underlying mechanisms. This study is important for rigorous assessment of PFAS bioaccumulation and their potential transfer along the food chain, which is important to assessing human exposure to PFAS and related health risks. Insights into PFAS uptake patterns in plants can inform management and mitigation strategies to minimize PFAS levels in edible parts of food crops and thus protect public health.

## 2 Materials and Methods

Data compilation, preprocessing, model training, evaluation, and interpretability analysis are described next and the workflow is shown in Supporting Figure S1.

### 2.1 Data Compilation

The data set was extracted from 13 peer-reviewed studies (Supporting Table S1) and included 2001 data points, covering diverse crop species, PFAS, soil properties, and cultivation conditions. Log-transformed BCF values (Log BCF) and PFAS concentrations (Log C) in plants were set as the output variables. The BCF values were obtained via two approaches: directly obtained from publications and calculated as the ratio of PFAS concentrations in plant tissues over those in soils (eq 1) for the studies without the BCF data (128 data points). Additionally, PFAS concentration data in plant tissues for two studies (184 data points) were extracted from plots in publications utilizing the software GetData Graph Digitizer (version 2.26.0, https://getdata-graph-digitizer.com/).

1 $$\mathrm{BCF}=\frac{C_{\mathrm{tissue}}}{C_{\mathrm{soil}}}$$

where *C*_soil_ represents the initial PFAS concentrations in soil and *C*_tissue_ is the PFAS concentrations in various tissue compartments of the plants. All concentrations in ng/g were reported on a dry-weight basis.

Four sets of variables, namely plant (species, tissues, categories, plant growth time, and presence of Casparian strip), soil properties (pH, soil organic matter [SOM], CEC, sand content, and initial PFAS concentration in soils), PFAS (compounds, molecular fingerprint, functional group, and carbon chain length) and cultivation condition (cultivation mode and additional PFAS input), were included as input features, as described in detail next. These 19 variables were chosen based on their relevance to plant uptake and their availability in the source literature (Supporting Table S2). Further, 11 of 13 studies were conducted in greenhouses and across the 10 of 13 studies that reported thermal data, mean daily temperature ranged from 16.3 to 28.5 °C (daytime temperature from 19.5 to 34.3 °C and nighttime temperature from 12.9 to 21 °C). This temperature range aligns with the common growing season conditions for major agricultural regions in temperate and subtropical zones. Importantly, within this data set, temperature variation is limited and noncontinuous, as it primarily reflects different controlled set points across greenhouse experiments rather than a natural environmental gradient. Given this limited and structured variability, temperature was excluded to avoid introducing an uninformative covariate. Soil water content was consistently held at 60–70% field capacity, thus plants were well watered without drought stress. Given that water availability plays a minor role in plant uptake when soil water is not a limiting factor, precipitation or irrigation water data are not included as input in model training. These covariates may be revisited once standardized, geo-referenced environmental data become available for extrapolating models to broader geoclimatic regions.

To ensure analytical consistency and precision, our models exclusively used the data for the samples from mature plants, thereby avoiding the variability associated with different plant growth stages and enhancing the model prediction performance and generalizability. Furthermore, plant growth time, which approximates the duration required for plants to reach maturity, captures the continuous exposure of plants to contaminated soils throughout the entire growth period. The data set includes 11 plant species (i.e., red chicory, lettuce, alfalfa, radish, carrot, celery, tomato, wheat, maize, sugar snap pea, and pea), 11 plant tissue compartments (where the shoot consists of stem and leaves), and 5 plant categories (leafy, root, fruit, cereal, and legume) based on edible parts or morphology (details in Supporting Table S3). The presence of a Casparian strip in plants is indicated by 1, while its absence is indicated by 0.

The data set has 21 PFAS (Supporting Table S4), including primarily the ones with carboxylate and sulfonate functional groups and a few with sulfonamide and other functional groups. Extended Connectivity Fingerprints (ECFP) with 1024 bits and a radius of 6 were adopted using the Morgan Algorithm implemented in the Python RDKit package. ECFP are circular topological molecular fingerprints that encode molecular structures into fixed-length binary bits. Specifically designed for structure–activity relationship modeling, ECFP capture detailed molecular substructures and have gained popularity in cheminformatics and machine learning modeling, due to their efficiency and reliability compared to physicochemical properties (e.g., *K*_ow_).^39,25^ Additionally, ECFP can be readily generated for new chemicals lacking physicochemical property data, thus enhancing model generalizability.^40^ More detailed information on ECFP is provided in Supporting Text S1.

The values of cultivation mode were set to 0 and 1 for the soil pot and field experiments, respectively. Additionally, in the experiments where plants were irrigated with PFAS-contaminated water during growth, the quantity of pollutants (μg) was determined and incorporated as an “additional input” variable. Given that all study sites were located away from potential contamination sources, such as Wastewater Treatment Plant (WWTP), landfills, and fluorochemical manufacturing facilities, foliar uptake was not explicitly quantified and was assumed to be minor.

### 2.2 Data Preprocessing

Given that the effectiveness of machine learning relies on the quality and relevance of the input data, data preprocessing is essential.^41^ It primarily involves assigning missing values, eliminating highly correlated variables, and converting categorical variables to numerical values (i.e., one-hot encoding). One-hot encoding converts categorical variables into a binary format by transforming each category into multiple binary columns, where a value of 1 indicates the presence of a particular category and 0 indicates its absence. This method treats each category as an independent entity, enabling a machine learning model to capture distinct patterns or behaviors of each category, and potentially enhance prediction performance. All categorical variables, including plant tissues, plant species, compounds, plant categories, and functional groups, were converted to one-hot encoding. This transformation was implemented using the *OneHotEncoder* function from the *Scikit-Learn* library. Missing data were handled via multiple imputations using the *IterativeImputer* class from Python’s *sklearn.impute* module. The data included many independent variables (e.g., molecular fingerprint bits) or multilinearly correlated variables. However, not all of the correlated features were significant predictors. In fact, the inclusion of redundant or nonsignificant features may lead to overfitting and reduce the applicability of a model. Hence, it is necessary to exclude those redundant features and only include the subsets of informative features within the original set.^42^ In this study, variables with zero variance were removed. For example, certain ECFP features that were uniformly zero across all PFAS compounds were deemed uninformative for model training, as they indicated the absence of corresponding substructures in all compounds. Consequently, these variables were removed. Additionally, when the Spearman correlation coefficient between two variables exceeded 0.8, the variable with the lower correlation to the target variable was discarded.

### 2.3 Machine Learning Models

Four widely used machine learning models were applied and evaluated in this study, including XGBoost, RF, MLP, and SVR. XGBoost is an efficient tree-boosting algorithm that iteratively adds trees to minimize a loss function, incorporating regularization to prevent overfitting.^43^ It supports parallel tree construction, enabling fast processing of large data sets. RF is an ensemble method comprising multiple decision trees.^44^ It generates trees using random subsets of data and features, aggregating their predictions through voting or averaging. RF is known for its robustness and ease of interpretation. MLP is a feedforward neural network with multiple layers of nodes. It captures complex relationships in data through interconnected neurons and is widely used for classification, regression, and pattern recognition.^45^ SVR is a robust regression methodology that leverages an ε-insensitive loss function to effectively manage data noise, thereby prioritizing predictions that fall within a predefined tolerance margin ε. Through the application of kernel functions, SVR transforms the input data into a high-dimensional feature space, enabling the model to adeptly capture intricate nonlinear relationships.^46^ To ensure uniformity in dimensions and units across all input variables, Z-score normalization was applied utilizing Python’s StandardScaler. Supervised learning was used because our data set is fully labeled and the prediction targets are continuous numerical values; unsupervised methods, by definition, are not suited for such predictive tasks. For a detailed introduction to these machine learning methods, please refer to Supporting Text S2.

### 2.4 Model Training and Evaluation

For model training, the data set was split into a training subset (72%), a validation subset (18%), and a testing subset (10%) via a two-step random split (first 90:10 for the test set, then 80:20 on the remainder for the training and validation sets). This approach ensures a held-out test set that does not participate in model training, with the subsequent marginal density histogram (Figure 1) confirming balanced distributions across subsets. The four machine learning algorithms mentioned above all require hyperparameter tuning to achieve the best prediction results on our data set. To optimize these four machine learning algorithms, grid search techniques based on K-fold cross-validation were employed to identify the optimal hyperparameters for the models. The hyperparameters of RF, XGBoost, MLP, and SVR were optimized separately, and the optimal combinations of hyperparameters were determined (Supporting Table S5), thereby ensuring that the performance evaluation of the models obtained through K-fold cross-validation on the training set was reliable and generalizable. This method involves dividing the training set into K subsets and using one of them as the validation set while the remaining ones serve as the training set in a rotating manner, thereby reducing the risk of high bias that may arise from a single train-test split. Considering the size of the data set in this study, 10-fold cross-validation was chosen for balance between computational efficiency and reliable performance estimation. After training the four machine learning models, the prediction performance of the models was assessed using the coefficient of determination (*R* ^2^), mean absolute error (MAE), and root-mean-square error (RMSE). *R* ^2^ represents the goodness of the model fitting, with the value close to 1 indicating a strong goodness of fit. The equations for MAE and RMSE are provided in Supporting Text S2.

**Figure 1:**
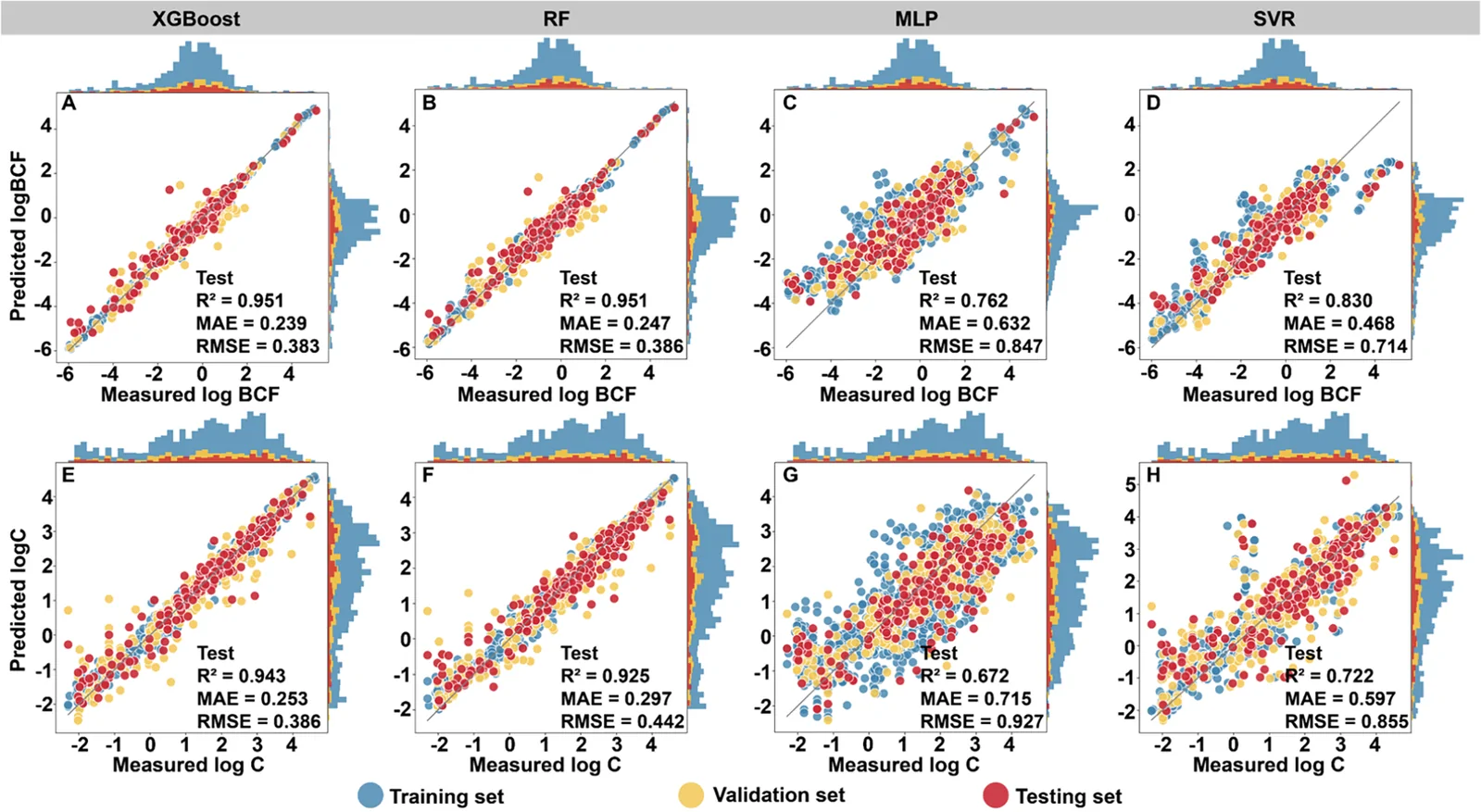
Measured and predicted Log bioconcentration factors (Log BCF) (A–D) and Log concentrations (Log C) (E–H) values with four machine learning models (XGBoost: eXtreme Gradient Boosting; RF: Random Forest; MLP: Multilayer Perceptron; SVR: Support Vector Regression). The density histograms on the top and right sides of each panel represent the measured and predicted values, with blue for the training set, yellow for the validation set, and red for the testing set.

### 2.5 Model Interpretation

#### 2.5.1 Feature Importance Assessment

We employed two methods, Permutation Feature Importance (PFI) and Shapley Additive exPlanations (SHAP), to quantify and interpret the contributions of individual feature variables to model predictions. PFI evaluates the significance of a feature by randomly permuting its values within the data set, thereby manipulating the original relationship between the feature and the target variable and subsequently comparing the model performance before and after permutation (details in Supporting Text S3). SHAP values, grounded in cooperative game theory, leverage Shapley values to elucidate the prediction outcomes of machine learning models, offering a robust and versatile framework for interpreting model predictions across a wide spectrum of complex models (details in Supporting Text S4). The Python toolkits employed for these methods include *scikit-learn* for PFI and the *TreeExplainer* module from the *SHAP* library.

#### 2.5.2 Partial Dependence Plot and Individual Conditional Expectation

Partial Dependence Plots (PDP) and Individual Conditional Expectation (ICE) curves are analytical instruments employed to decipher the relationship between features and the target variable within machine learning models. The partial dependence of the predictions on the feature was explored by continuously varying its value while holding other features constant. PDP is a global interpretation method that averages the contributions of all other features to reveal the overarching influence of the feature of interest in the target variable. ICE, in contrast, illustrates how predictions for specific samples change with variations in a particular feature, offering insights into individual sample responses. PDPs can also be utilized to delineate the joint impact of multiple features on predictions, showcasing the model’s behavior under various combinations of these features. In our analysis, we strategically fixed one feature’s value to simplify the examination, thereby isolating the effects of specific combinations of the remaining two features on the model’s predictions. This approach allows for a more focused investigation of feature interactions while preserving the model’s predictive complexity. All PDPs and ICE curves were completed using the *scikit-learn* library embedded in Python 3.12.

## 3 Results and Discussion

### 3.1 Model Performance

All the machine learning models showed satisfactory predictive performance (Figure 1 and Supporting Table S6) with *R* ^2^ of 0.762–0.951, MAE of 0.239–0.830, and RMSE of 0.383–0.847 for Log BCF and *R* ^2^ of 0.672–0.943, MAE of 0.253–0.751, and RMSE of 0.386–0.927 for Log C, respectively.

Among these four machine learning models, the XGBoost model has the best performance for both Log BCF (*R* ^2^ = 0.951, MAE = 0.239, RMSE = 0.383; Figure 1A) and Log C (*R* ^2^ = 0.943, MAE = 0.253, RMSE = 0.386; Figure 1E), followed by the RF model (Figure 1B,F). Learning curve and early stopping analyses showed no signs of overfitting (Supporting Figure S2). MLP and SVR models have relatively lower predictive performance, with *R* ^2^ < 0.84, MAE > 0.46, and RMSE > 0.71. A detailed comparison of the algorithmic characteristics underlying these performance differences is provided in Supporting Text S2. Because soil PFAS concentrations spanned more than 5 orders of magnitude (0.025 to 10,581 ng/g), we evaluated whether high concentration samples disproportionately influenced model performance. Stratified test-set results showed strong performance across all four concentration intervals (Table S7). Despite moderately higher errors below 10 ng/g, the Log C and Log BCF models achieved *R* ^2^ values of 0.881 and 0.883, respectively, indicating that the overall satisfactory performance across all soil PFAS concentration ranges.

Previous studies have consistently shown that gradient boosting and other decision tree-based models generally exhibit superior predictive performance in estimating BCF/BAF. Xiang et al.^31^ observed that the gradient boosted regression trees (GBRT) achieved a higher *R* ^2^ of 0.85 than RF (0.81) and SVR (0.62) for predicting root concentration factor (RCF) in soil-based environments. Adu et al.^32^ found that the RF model (*R* ^2^ = 0.77) outperformed the support vector machines (SVM) and artificial neural networks (ANN) models for predicting RCF in hydroponic environments. Similarly, Song et al.^38^ demonstrated that the XGBoost exhibited the highest prediction performance for Log BAF, achieving an R^2^ of 0.90 and RMSE of 0.40. Compared to prior studies, our XGBoost and RF models achieved higher *R* ^2^ and lower RMSE. This improvement is largely attributable to methodological upgrades: (i) a 2.5- to 7-fold larger data set (*n* = 2001); (ii) stricter data-quality control, including removing highly correlated variables and constraining plant samples to a consistent growth stage.

### 3.2 Identification of the Important Features

PFI and SHAP analyses were conducted to identify the most important features influencing the Log BCF and Log C values of PFAS, based on the XGBoost model. Detailed PFI and SHAP values for each feature are presented in Supporting Table S8.

#### 3.2.1 Important Features for Log BCF

PFI and SHAP consistently ranked the same top 10 predictors of Log BCF, including three soil features (pH, CEC, and initial PFAS concentration in soil), one chemical structure (carbon chain length), and six plant features (four specific tissues, one species, and growth time) (Figure 2A–C). Soil pH value and carbon chain length of PFAS were the top two important features (PFI scores ≈ 1.63 and 1.00, SHAP value contributions ≈ 31% and 13%). Compared with these two features, specific plant tissue (root), plant species (red chicory), as well as CEC showed relatively less influence on the Log BCF values, with PFI scores of 0.64, 0.41, and 0.37. The other features (e.g., leaf tissue, initial PFAS concentration in soil) showed a minor influence, with a relatively small importance (PFI score <0.24). Our results further support the important role of soil pH on PFAS uptake by plants as reported previously.^31,38^ Furthermore, PFAS carbon chain lengths stand out as a key predictor, despite that molecular weight and molecular size have also been shown to be important.^33,31,38,32^ Consistent with previous reports that plant species strongly governs PFAS accumulation,^32^ our model further reveals that root and leaf tissues make the largest species-specific contributions. The ranking highlights the collective control of soil chemistry, PFAS structure, and plant traits on PFAS accumulation in plants. Detailed mechanistic interpretation of these drivers is provided in Section 3.3.1.

**Figure 2:**
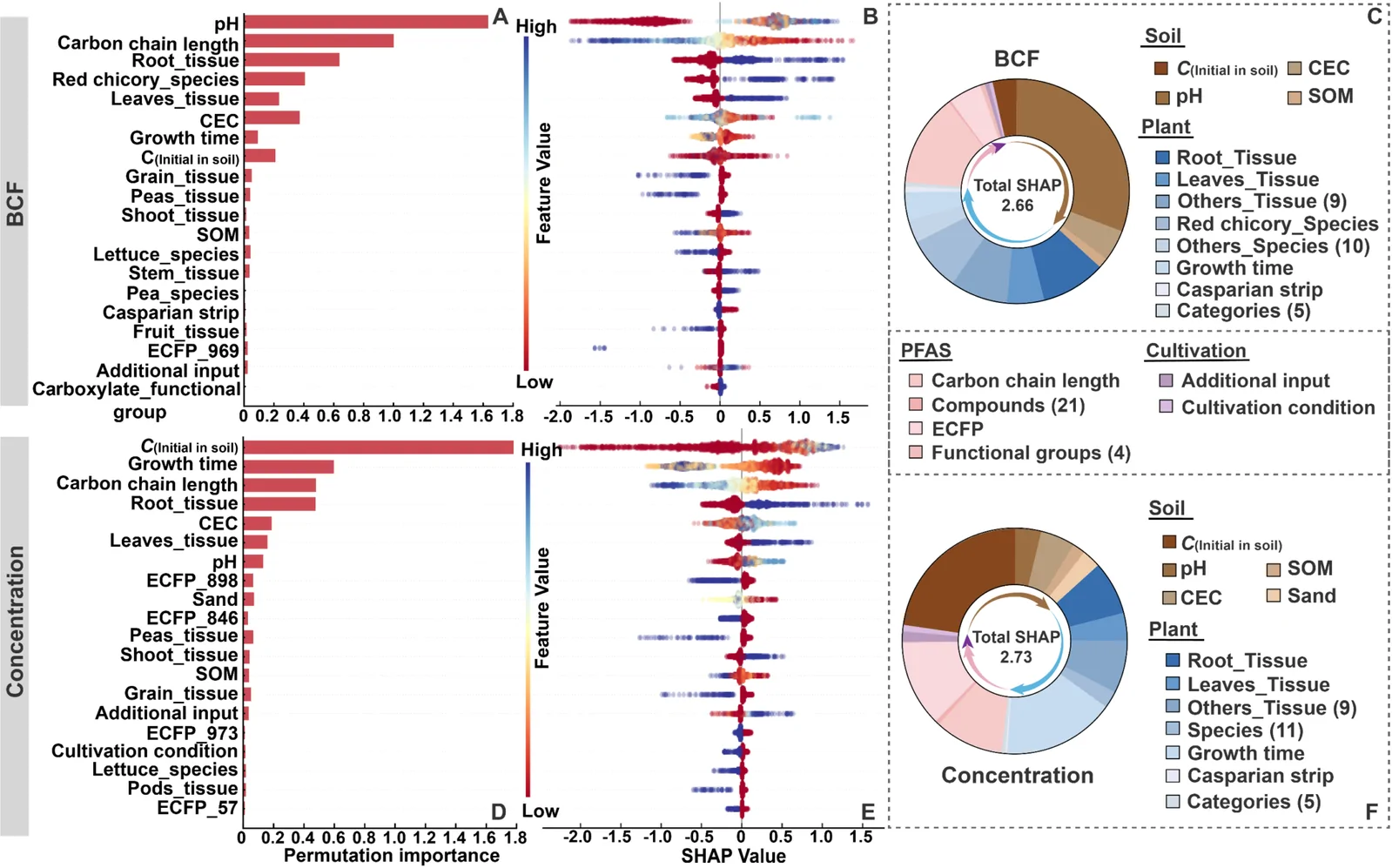
Analysis of permutation feature importance (PFI) (A, D); SHapley Additive exPlanations (SHAP) summary plot illustrating the influence of individual features on predictions (B, E); average SHAP value for all individual features on predictions (C, F); the substructures represented by important extended connectivity fingerprints (ECFP) bits are illustrated in Supporting Table S9.

#### 3.2.2 Important Features for Log C

PFI and SHAP converged on the same six leading predictors of Log C (Figure 2D–F): soil PFAS concentration, plant growth time, carbon chain length, plant root, CEC, and plant leaves. Despite absence among the top drivers of Log BCF, the initial concentration of PFAS in soils dominated (PFI = 1.80; SHAP contribution ≈ 23%), highlighting a concentration-dependent accumulation pathway that will be mechanistically discussed in Section 3.3.2. Plant growth time was the second most significant factor, with PFI = 0.60 and SHAP contribution = 16% (mean absolute SHAP = 0.44), followed by carbon-chain length (PFI = 0.48) and root tissue (PFI = 0.48). Other factors, including CEC, plant leaves, and soil pH, exhibited relatively modest effects (PFI = 0.13–0.19).

### 3.3 Influence of the Important Features on the Prediction Values

Using ICE and PDP, we quantify how the key drivers identified in the above section modulate the predicted Log BCF and Log C. All partial-dependence profiles were compared with SHAP to ensure robustness. Mechanistic interpretation is provided separately for Log BCF and Log C, respectively.

#### 3.3.1 Feature Analysis for Log BCF

Soil pH emerges as the most critical factor influencing Log BCF values. As depicted in Figure 3A, between pH 5.5 and 6, the average SHAP values transition abruptly from negative to positive and the ICE plots also reveal a sudden increase. Above pH 6, BCF values are oscillatory but remain elevated. Recently published machine learning models reported similar peaks at pH 6–7, followed by a rapid decrease until pH 7.7.^31,38^ This pattern may result from the pH-dependent speciation of PFAS, especially ionizable compounds. Most of the PFAS in this study are carboxyl- and sulfonate-containing compounds, with p*K*_a_ < 1 (Supporting Table S4), which means their anionic form predominates across the entire soil pH range. Both these PFAS and soil colloid surfaces (clay and SOM surfaces) are overall negatively charged at higher pH, thus increasing electrostatic repulsions between PFAS and soil surfaces and reducing PFAS sorption to soils.^16,47^ For positively charged sites from iron and aluminum oxide patches or aluminum oxides in clay edges, increasing soil pH also lowers the average positive charges, thus decreasing electrostatic attraction with anionic PFAS. Lower PFAS sorption to soils increases dissolved PFAS in soil water, therefore enhancing plant availability and uptake.^27,48^ However, the speciation of perfluorooctane sulfonamide (PFOSA, p*K*_a_ ∼ 6.24) is highly pH-dependent.^49^ At pH < p*K*_a_, it remains predominantly a neutral molecule with high hydrophobicity (Log *K*_oc_ = 4.1 cm^3^/g), favoring sorption to SOM.^47,50^ As pH rises above ∼ 6.24, PFOSA progressively deprotonates to an anion, resulting in electrostatic repulsion with negatively charged soil surfaces.

**Figure 3:**
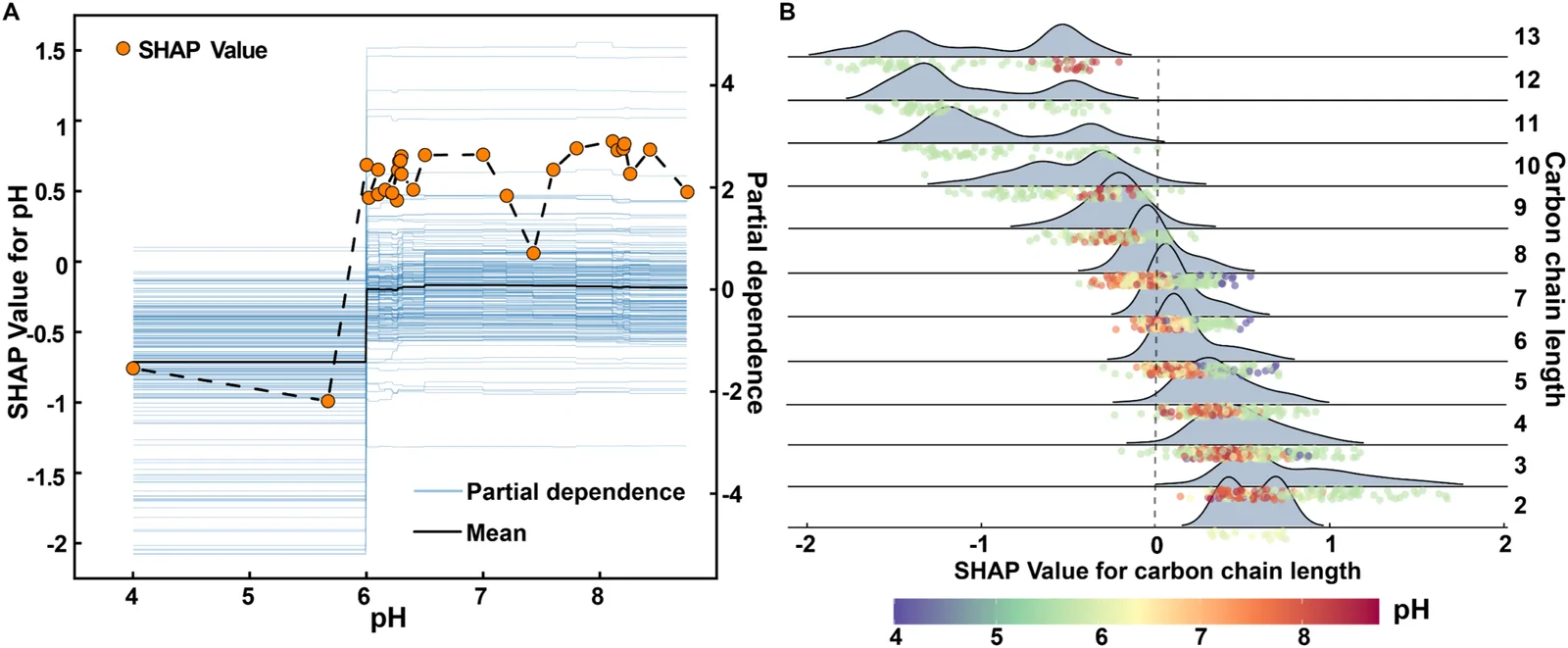
Partial dependence and SHAP distributions. (A) SHAP values, partial dependence line (black) and individual conditional expectation (ICE) curves (blue) for pH; (B) kernel-density heatmap of SHAP value within each carbon chain length bin, with dot color indicating the corresponding soil pH.

Additionally, soil pH affects plant growth and related physiological processes, thereby influencing PFAS absorption capacity. For example, the optimal pH range for wheat is 5.8–6.5, and a hydroponic study revealed that the uptake and accumulation of PFOS by wheat were maximized at solution pH of 6.^51^ Peak accumulation occurs under near-neutral conditions (pH ≈ 6), consistent with optimal plant vigor. Moreover, pH may affect PFAS fate through plant rhizosphere processes. At low soil pH, roots up-regulate plasma membrane H^+^-ATPase activity to maintain cellular homeostasis.^52^ This enhanced H^+^-ATPase activity can promote the transport and translocation of certain PFAS across root cell membranes, as it establishes the proton motive force that powers secondary active transport.^53^ However, the increased extrusion of H^+^ also acidifies the rhizosphere locally, which enhances PFAS sorption to soil particles and root cell walls, thereby reducing the freely dissolved concentration available for root uptake. Consequently, although H^+^-ATPase activity is upregulated, the overall accumulation in plants is diminished under low pH conditions, as the reduced bioavailability at the root–soil interface outweighs the enhanced internal transport capacity. Beyond proton pumps, pH stress can also alter the composition and quantity of root exudates,^54^ a complex mixture of organic acids, sugars, and humic-like substances, that can influence PFAS fate through multiple pathways. For example, root exudates may induce microbial expression of defluorination enzymes and thereby promote the biotransformation of certain PFAS.^55^ Among these exudates, low-molecular-weight organic acids (LMWOAs), such as citrate, oxalate, and malate, may further influence PFAS partitioning in the rhizosphere. For example, LMWOAs may compete for mineral sorption sites with PFAS and modify mineral surface properties, thus facilitating PFAS desorption from soils, increasing dissolved PFAS concentrations, and potentially enhancing root uptake.^56,57^ The rhizosphere-mediated processes likely act in concert with the direct effects of soil pH on PFAS speciation and soil surface charges. Thus, the impact of soil pH on PFAS accumulation in plants results from both physicochemical effects (e.g., PFAS speciation, soil surface charges, and electrostatic interactions) and plant-mediated biological effects (e.g., H^+^-ATPase activity and root exudation).

SHAP values decline monotonically with carbon-chain length (Figure 3B), illustrating an overall negative correlation between Log BCF and carbon-chain length and confirming preferential accumulation of short-chain PFAS (see also ICE/PDP plots in Supporting Figure S3). This highlights the necessity of incorporating carbon chain length as a critical parameter when evaluating the environmental behavior and phytotoxic potential of PFAS. Further, root tissue exhibits the lowest absolute SHAP values for carbon chain length (Figure 4A), indicating less chain-length preference. Above-ground parts (e.g., leaves, shoots, stems, and peas) display steep downward trends of SHAP values that underscore pronounced chain-length selectivity. Plants take up PFAS in soil pore water through roots, in which dissolved PFAS may pass through root membranes via either apoplastic or symplastic pathways before reaching xylem where they may be translocated to aboveground compartments via transpiration.^58^ Short-chain PFAS, characterized by lower hydrophobicity and smaller molecular weights, are more readily taken up and translocated by plant roots, exhibiting higher BCF in plant tissues. In contrast, with high *K*_ow_ and large molecule size, long-chain PFAS are more strongly sorbed by soil particles and/or retained by biological barriers during transfer in plants, thus limiting their bioavailability and accumulation.^9,18,59^ It has been widely reported that the efficiency of PFAS translocation declines markedly with chain length.^60^ Long-chain PFAS exhibit a higher binding affinity to apoplast/symplast tissue in roots (e.g., lipid, protein) and slower mobility,^20,61^ and their passage across biological barriers (e.g., the Casparian strip) is increasingly restricted.^12,14,62^ Therefore, although roots may acquire high concentrations of long-chain PFAS, only a small fraction migrates into the xylem, resulting in substantially lower BCF in above-ground tissues.

**Figure 4:**
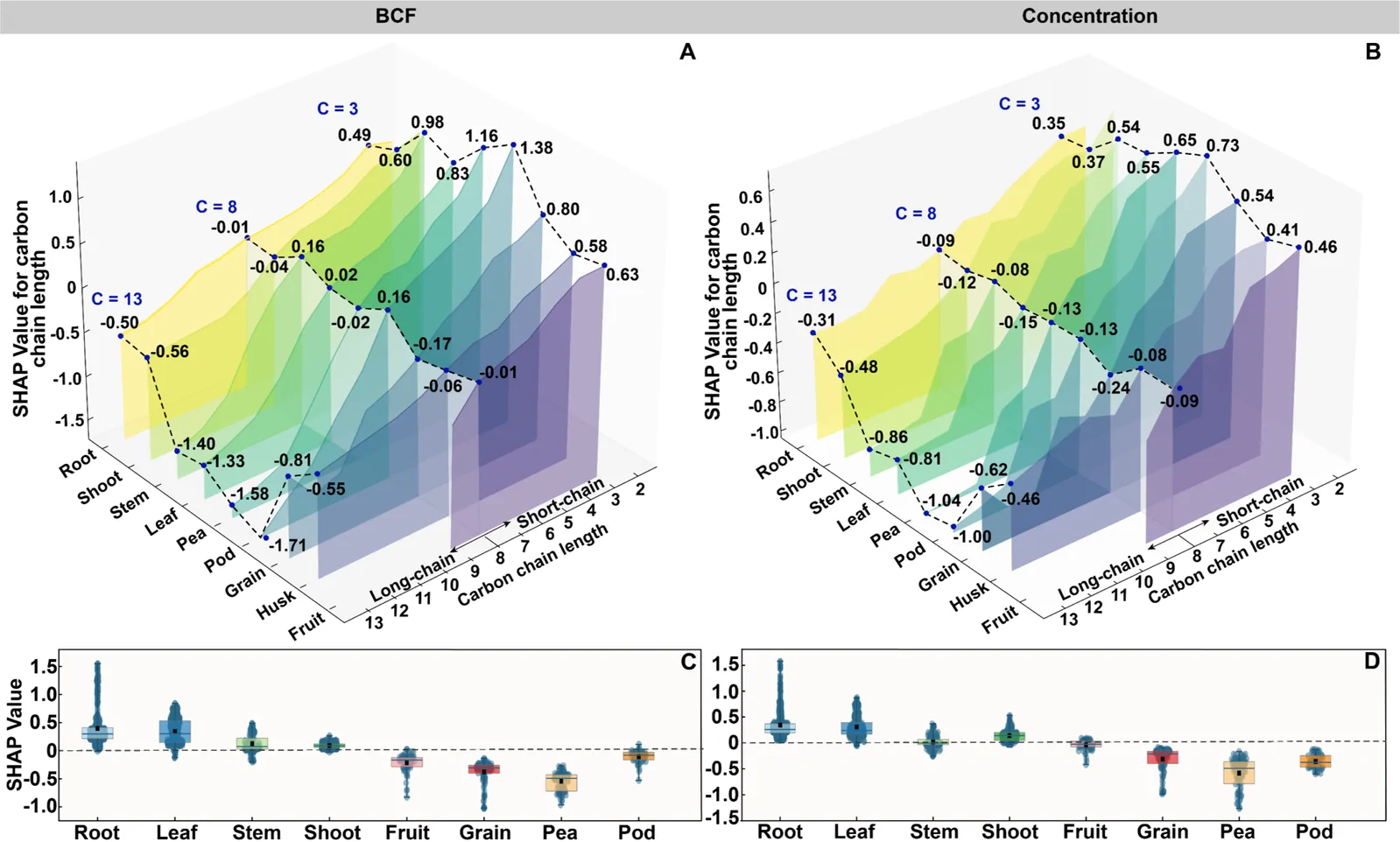
SHAP value of carbon chain length with interaction effect of “plant tissues” in the Log BCF model (A) and the Log C model (B); SHAP value of various plant tissues in the Log BCF model (C) and the Log C model (D).

SHAP values reveal a clear tissue-specific hierarchy of PFAS accumulation (Figure 4C): roots and leaves exhibit the highest mean absolute SHAP values (0.39 and 0.34), followed by stems (0.12) and shoots (0.09), while peas, grains, and fruits display negative SHAP values, indicating contributions that reduce predicted BCF. This result is consistent with previous studies, which reported that PFAS tend to accumulate more in the vegetative compartments of plants, such as roots, stems, and leaves, compared to storage organs like fruits, grains, and peas.^63,11^ Species-level analysis (Supporting Figure S4) further revealed more complex, nuanced understanding on the prevailing notion of an inverse relationship between BCF and carbon chain length in roots. In fact, BCF in roots exhibited a U-shaped pattern with increasing chain length in lettuce, radish, pea, wheat, and maize, i.e., maximal accumulation for both very short-chain (C ≤ 4) and long-chain PFAS (C ≥ 9). While leaves and shoots preferentially enriched short-chain PFAS, roots served as the principal sink for long-chain PFAS, underscoring the need for tissue- and species-specific dietary exposure and risk assessment.

Low soil pH amplifies the chain-length effect in above-ground tissues. The SHAP interaction plot of carbon chain length and pH (Figure 3B) shows larger absolute SHAP values under low pH conditions, indicating a stronger carbon chain length dependence, compared to high pH conditions. Consistently, the 3D interaction plot of carbon chain length, pH, and tissues (Figure 5) reveals that for parts such as shoots and leaves, the BCF for long-chain PFAS shows a significant decrease when pH is below 6. Under low pH conditions, where plant transpiration is not suppressed, the transpiration-driven PFAS transport may amplify the intrinsic chain-length discrimination. Conversely, under alkaline soil conditions transpiration is likely constrained,^64–66^ and therefore decreases the preferential accumulation of short-chain PFAS, resulting in a more uniform PFAS distribution across plant tissues. Beyond the 3D surface plots shown in Figure 5, all other variable combinations did not reveal similarly strong or nonmonotonic interaction patterns. Therefore, no additional 3D PDPs are presented.

**Figure 5:**
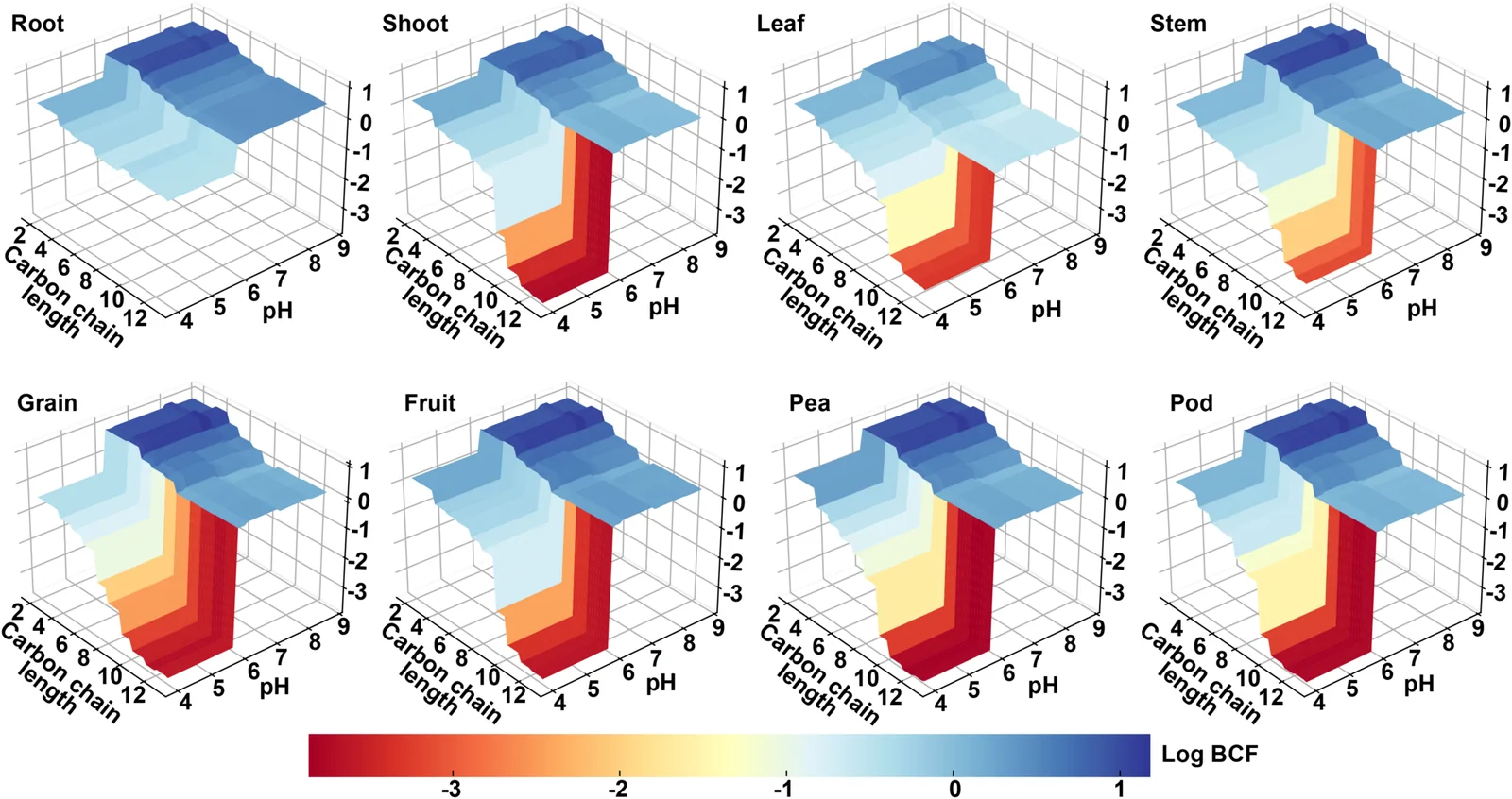
Interaction impacts of carbon chain length-pH-tissues on the BCF values.

Soil CEC was also identified as a relevant feature for BCF. The SHAP summary plot (Figure 6A) shows that an overall negative correlation between CEC and the predicted Log BCF, suggesting that higher soil CEC generally corresponds to lower plant uptake of PFAS, primarily because enhanced PFAS sorption to soils and thus reduced PFAS bioavailability.^27,67^ CEC correlates positively with both clay and SOM content, which are key sorbents for PFAS. Increasing SOM can strengthen hydrophobic interaction, particularly for the long-chain PFAS,^27^ while clay can contribute to PFAS sorption through the direction sorption of PFAS by positively charged aluminum oxide in clay edges or cation bridging of PFAS with negatively charged sites.^68^ Moreover, the high surface area of clay minerals can promote van der Waals interactions and provide more sorption sites. Consequently, higher CEC may enhance overall soil sorption capacity of PFAS through a combination of mechanisms, including cation bridging, electrostatic attraction, and hydrophobic interactions, ultimately reducing plant uptake of PFAS.

**Figure 6:**
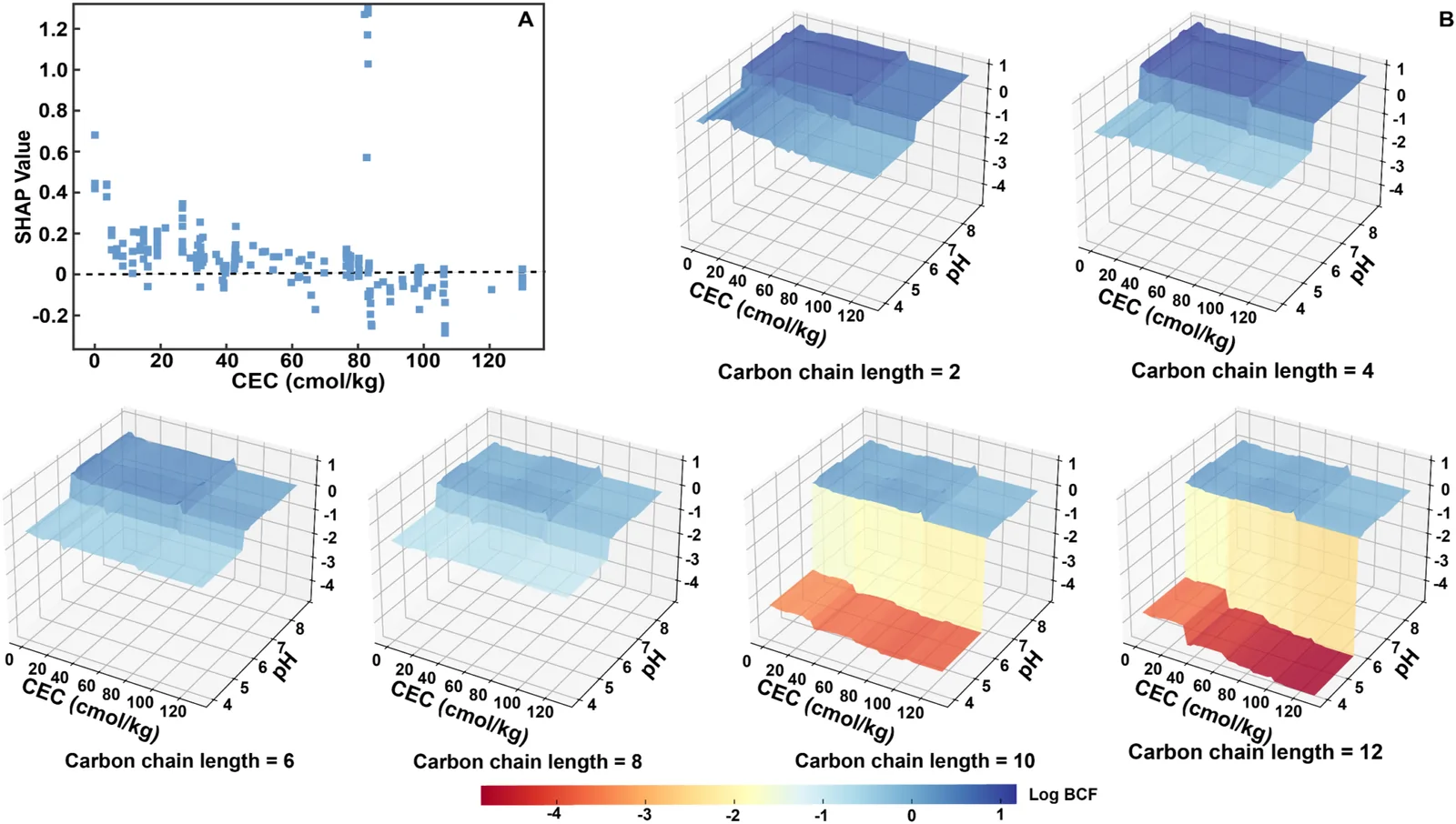
SHAP value of cation exchange capacity (CEC) in the Log BCF model (A); Interaction impacts of carbon chain length-pH-CEC on the BCF value (B).

Given the intrinsic correlation between CEC and pH, we further investigated their interactive effects. The 3D PDP (Figure 6B) reveals a clear, synergistic pattern that is consistent across all carbon chain lengths (shown for a selection of PFAS for clarity). The model predicts the Log BCF is minimized under conditions of high pH and low CEC but maximized under low pH and high CEC. Consequently, soils with high pH and low CEC, such as calcareous sandy soils, Calcaric Arenosols and sandy Calcisols, are predicted to have the lowest accumulation risk. In contrast, soils with low pH and high CEC, such as organic-rich Histosols (peat soils), Umbrisols, and acidic high-activity clay soils such as Alisols, may represent conditions with the highest predicted plant accumulation risk.

The high feature importance of red chicory suggests its potential as a PFAS hyperaccumulator. While the underlying mechanisms of elevated PFAS uptake by red chicory remain to be elucidated, it has been identified as a strong accumulator of cadmium, exhibiting high biologic absorption coefficients (BAC > 3) and translocation factors (TF > 5), and a moderate accumulator of zinc (TF > 3 but BAC < 1).^69^ The large BAC and TF values signify an efficient root uptake and xylem transport system, which may also facilitate PFAS uptake and translocation. Therefore, the prominence of red chicory in our model highlights the need of examining its PFAS residues for improving food safety and its potential for phytoremediation, which could be further investigated in future experiments.

#### 3.3.2 Feature Analysis for Log C

The initial concentration of PFAS in soil was identified as the most critical predictor of PFAS accumulation in plants. The ICE and PDP plots show a steep initial increase in the predicted Log C, followed by leveling-off at higher soil PFAS concentrations (Supporting Figure S5). This pattern is directly reflected in the species-specific data, where PFAS concentrations across all crops increase rapidly at low levels of initial soil PFAS concentrations before reaching a plateau (Figure 7A). This initial concentration-dependent increase was reported by previous studies.^11,70^ It is known that the uptake and transport of contaminants by plants involves passive diffusion, active transport, and anion and aquaporin channels.^18,19,71^ Active transport is an active process mediated by carrier proteins, whereas recent kinetic studies indicate that even the passive process of PFAS influx could be carrier-mediated.^72,73^ Although these pathways may operate concurrently and their relative contributions may vary with PFAS chain length, plant species, and environmental conditions,^73,74^ they all depend on a finite pool of membrane transporters, and/or entry channels. At low soil concentrations these sites are far from saturation, so PFAS uptake increases with soil PFAS concentration. As soil PFAS concentration increases, the available PFAS overwhelms the transport capacity and the PFAS uptake reaches a plateau.^72,73^

**Figure 7:**
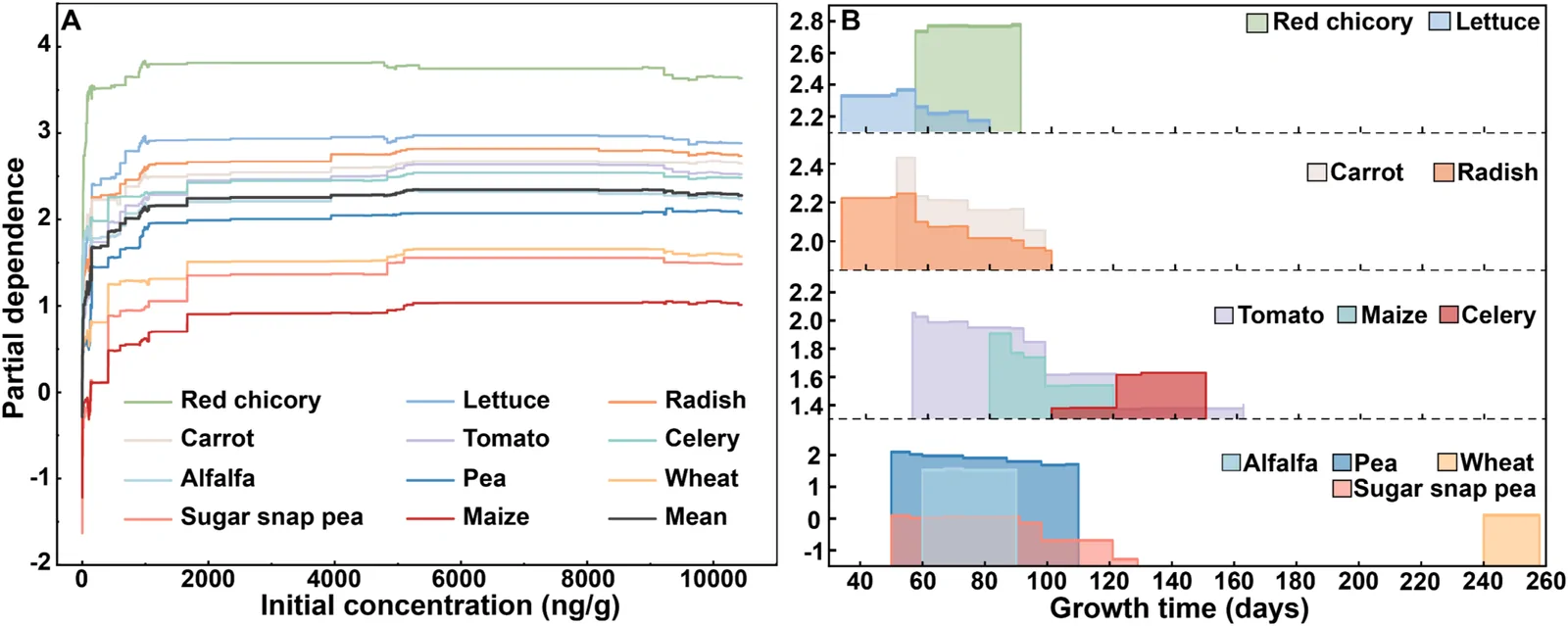
Partial dependence plot of initial concentration in soil (A) and growth time (B) for the Log C model.

The model reveals an overall negative relationship between the plant growth time and the predicted PFAS concentration (Supporting Figure S6). Shorter-cycle species (e.g., red chicory, lettuce, and radish) accumulated higher PFAS concentrations than the longer-cycle (e.g., corn, celery, wheat). To model this relationship explicitly, we leveraged maturity-day data for various cultivars across 11 species to model the average PFAS concentrations in all sampled tissues at harvest (Figure 7B). This negative maturity–concentration relationship was also suggested among cultivars within several species. This empirical observation is consistent with (i) their intrinsically higher relative growth rate and more vigorous metabolism, and (ii) their inherently smaller whole-plant biomass for shorter-cycle species. The fast-growing trait results in elevated nutrient demand and consequently greater root uptake capacity.^75^ Empirical evidence shows that high relative growth rate and vigorous root activity significantly enhance the uptake of PFAS.^76,77^ Additionally, their reduced biomass limits the dilution effect of expanding biomass. For example, McDonough et al.^60^ observed higher PFAS concentrations in smaller individual fruits despite more cumulative PFAS mass in larger fruits. However, we acknowledge that this association may be partially confounded by species types, and validation at species level would be needed.

Figure 7 also reveals that several plants with higher PFAS concentrations belong to leafy (red chicory and lettuce) and root vegetables (carrot and radish), which is consistent with the BCF result. This finding aligns with previous experimental evidence demonstrating that PFAS preferentially accumulate in leafy vegetables.^78^ Similar to its effect on Log BCF, PFAS carbon chain length also displays an overall negative correlation with the predicted Log C (Supporting Figure S7), with a parallel pattern of differential accumulation across plant parts. Although the carbon chain length effect on PFAS absorption by plant roots is not pronounced (Figure 4B), the concentration in plant roots is indeed the highest (Figure 4D), indicating that plant roots have a strong absorption capacity with less selectivity based on chain length. The accumulation concentration of PFAS in plant leaves is comparable to that in roots. For short-chain PFAS, their concentrations in leaves are slightly higher than those in roots (Figure 4B). However, due to the pronounced carbon chain length effect, the accumulation of long-chain PFAS in leaves decreases and falls below that in roots. Overall, the concentrations in roots and leaves are higher than in other parts, followed by shoot, stem, and fruit. For edible parts of crops, the lowest PFAS concentrations were in grains and peas. This result is consistent with experimental evidence, such as Shen et al.,^78^ reporting that PFAS concentrations were the lowest in grains and were significantly higher in root vegetables, followed by leafy, stem, and fruit vegetables with no significant difference.

## 4 Implications

This study has successfully applied and trained machine learning models with high predictive performance for PFAS uptake in plants, which is important for assessing dietary exposure and potential health risks associated with PFAS. Our results showed that soil pH, carbon chain length, and plant-specific tissues are the most significant predictors of BCF. Our models predicted elevated plant uptake of PFAS occurs at soil pH greater than 6. Thus, for PFAS-contaminated soils, it may be possible to limit crop uptake of PFAS by controlling soil pH slightly less than 6.0 (e.g., pH 5.5–6.0), for example, acidifying soils with elemental sulfur or acid-forming fertilizers. However, this approach must be carefully evaluated on a crop-specific basis, as altering pH outside a crop’s optimal range can adversely affect crop yield. While carbon chain length of PFAS is on average negatively correlated with their accumulation in plants, PFAS uptake by roots is less sensitive to carbon chain length, comparing to above-ground parts such as leaves that preferentially accumulate short-chain PFAS. Thus, both long-chain and short-chain PFAS should be examined and managed in edible roots of crops, whereas attention could be directly more to short-chain PFAS in above-ground plant tissues. Particularly at soil pH < 6, PFAS uptake in above-ground tissues is strongly influenced by carbon chain length, indicating acidic soils allow for suppression of plant uptake of long-chain PFAS that includes the ones with well-recognized toxicity to humans (e.g., PFOA and PFOS).

Further, this study underscores the critical role of soil PFAS concentration as the primary driver of PFAS accumulation in plants, evidenced by an initial sharp rise in plant PFAS concentrations before leveling-off with increasing soil PFAS concentrations. Clearly, if PFAS contamination is a concern in agricultural soils, testing for PFAS in soils may be needed and a threshold of soil PFAS concentrations may be established for consideration of soil remediation. The observed association between growth time and PFAS accumulation suggests that selection of longer-cycle cultivars or crop species may warrant further investigation as a potential mitigation strategy, providing that within-species and cross-species validations are conducted. It is also important to consider the PFAS residue levels in edible parts of various crops and the typical consumption rates to reduce human exposure and related health risks. In addition, leafy and root vegetables exhibit higher PFAS concentrations, aligning with their observed higher BCF; within the plant, roots and leaves accumulate the most PFAS, with grains and peas containing the least. Therefore, at PFAS-contaminated fields, production of leafy greens and root vegetables should be avoided, if possible, whereas grain and pea crops may be grown to minimize the PFAS levels in edible portion of food crops.

This study revealed fundamental insights on the effect of soil conditions, PFAS properties, crop varieties, and plant tissues on PFAS uptake and accumulation in food crops and illustrates how this knowledge could help develop mitigation and remediation strategies to minimize PFAS exposure through the food chain. It is noted that this study is limited in the availability of experimental data including 21 PFAS compounds, 11 crop species, and a narrow mean daily temperature range of 16.3 to 28.5 °C, mimicking the growth condition in temperate and subtropical climatic zone. As more experimental data become available, covering more diverse crop types, PFAS compounds, and geoclimatic conditions in the future, the models could be further improved. Additionally, the root PFAS concentrations compiled from the literature reflect total tissue burdens and do not differentiate between surface adsorbed and intracellularly absorbed fractions. This distinction is analytically challenging and rarely addressed in routine studies. Future work incorporating fraction-specific measurements would further improve mechanistic understanding of root uptake. Furthermore, because experimental data can be limited, one potential strategy may be to define the model’s applicability domain and explore ways to model crop uptake of PFAS in data-scarce scenarios, for example, for “unseen PFAS, crops, or soils”, by leveraging prowess of physics-infused or knowledge guided machine learning, e.g., integration of mechanistic soil-crop models (e.g., Soil-Water-Atmosphere-Plant model [SWAP][Bibr ref79]) with machine learning. Such integration of machine learning with prior knowledge and mechanistic processes could help develop models that overcome the data scarcity and allow for scenario analysis to support management decision for minimizing PFAS residues in food crops.

## Supplementary Material



## Data Availability

All models were implemented in Python 3.11.0, utilizing standard machine learning libraries including scikit-learn and XGBoost for modeling, and SHAP for interpretability analysis. The complete source code, data set, requisite environment details, and Jupyter notebooks to reproduce all results are publicly available in the project’s GitHub repository: https://github.com/Rui488/BCF-Concentration-of-PFAS-in-plant.git.

## Notes

Extended circular fingerprint (ECFP) (T1); Machine Learning Models (T2); permutation feature importance (PFI) (T3); Shapley Additive exPlanations (SHAP) (T4); List of the 13 studies used for data set compilation (Table S1); all input variables for model prediction (Table S2); the category, species and tissue of plant collected in the data set (Table S3); the name and structures of PFAS collected in the data set (Table S4); hyperparameters for the machine learning procedure (Table S5); performance metrics of four machine learning models (XGBoost, RF, MLP, SVR) on Log BCF and Log C prediction (Table S6); performance of the Log C and Log BCF models across soil PFAS concentration ranges in the independent test set (Table S7); Feature Importance Scores from PFI and SHAP (Table S8); the important substructures affecting BCF and concentration of PFAS (Table S9); referential logical framework for predicting plant uptake of PFAS based on machine learning model (Figure S1); learning curves and early stopping analysis for the XGBoost models (Figure S2); individual conditional expectation (ICE) and partial dependence plot (PDP) of PFAS carbon chain length on Log BCF (Figure S3); ICE and PDP of PFAS carbon chain length on Log BCF in various plant tissues (Figure S4); ICE and PDP of initial concentration in soil on Log C (Figure S5); ICE and PDP of plant growth time on Log C (Figure S6); ICE and PDP of PFAS carbon chain length on Log C (Figure S7) (PDF)
