# Supplementary material for: Predicting Bioconcentration Factors and Concentrations of Per- and Polyfluoroalkyl Substances in Plants with Machine Learning
Source: Environ Sci Technol. 2026 Sep 1;60(36):25786–99. doi: 10.1021/acs.est.6c10458 (PMC13585049; doi:10.1021/acs.est.6c10458)
Supplement: Supplementary file 1 [file es6c10458_si_001.pdf]

Supporting Information for

Predicting Bioconcentration Factors and Concentrations of Per- and Polyfluoroalkyl Substances in Plants with Machine Learning

Rui Li [a], Jiyoon Yi [b], Hui Li [a], Wei Zhang [a,c*]

[a] *Department of Plant, Soil and Microbial Sciences, Michigan State University, East Lansing, Michigan 48824, United States*

[b] *Department of Biosystems and Agricultural Engineering, Michigan State University, East Lansing, Michigan 48824, United States*

[c] *Environmental Science and Policy Program, Michigan State University, East Lansing, Michigan 48824, United States*

---

[*]**Corresponding author.**

Address: Department of Plant, Soil and Microbial Sciences, Michigan State University, East Lansing, Michigan 48824, United States. Tel: +1517-353-0471. Fax: 517-355-0270. *E-mail address*: weizhang@msu.edu

Supplementary materials include 26 pages with 4 texts, 9 tables, 7 figures and 1 reference list.

Texts **1**

Text S1: Extended circular fingerprint (ECFP) 1

Text S2: Machine Learning Models 2

Text S3: Permutation Feature Importance (PFI) 4

Text S4: Shapley Additive exPlanations (SHAP) 5

Tables **6**

Table S1 List of the 13 studies used for data set compilation 6

Table S2: All input variables for model prediction 7

Table S3: The category, species and tissue of plant collected in the data set 8

Table S4: The name and structures of PFAS collected in the data set 9

Table S5: Hyperparameters for the machine learning procedure 11

Table S6: Performance metrics of four machine learning models (XGBoost, RF, MLP, SVR) on Log BCF and Log C prediction 12

Table S7: Performance of the Log C and Log BCF models across soil PFAS concentration ranges in the independent test set 13

Table S8: Feature Importance Scores from PFI and SHAP 14

Table S9: The important substructures affecting BCF and concentration of PFAS 15

Figures **16**

Figure S1: Referential logical framework for predicting plant uptake of PFAS based on machine learning model 16

Figure S2: Learning curves (A-D) and early stopping (E-F) analysis for the XGBoost models. R^2, coefficient of determination; RMSE, root mean square error 17

Figure S3: Individual conditional expectation (ICE) and partial dependence plot (PDP) of PFAS carbon chain length on Log BCF 18

Figure S4: ICE and PDP of PFAS carbon chain length on Log BCF in various plant tissues 19

Figure S5: ICE and PDP of initial concentration in soil on Log C 20

Figure S6: ICE and PDP of plant growth time on Log C 21

Figure S7: ICE and PDP of PFAS carbon chain length on Log C 22

References **23**

Text S1 Extended circular fingerprint (ECFP)

ECFP (Extended Connectivity Fingerprints) are circular topological fingerprints designed to capture specific substructures within a molecule, explicitly accounting for various bond types, including single (sp3-hybridization), double (sp2-hybridization), triple, and aromatic bonds.[1] Initially, an integer identifier is assigned to each atom of a molecule, based on its atomic properties, including atomic number, atomic mass, atomic charge, valence, the number of attached hydrogens and a flag indicating ring membership. These properties are hashed into a 32-bit integer value, serving as the initial atom identifier.

In the iterative updating phase, each atom collects and sorts the identifiers of its immediate neighbors, combines them into an array, and hashes this array into a new identifier, which replaces the old one. This iteration process is repeated several times. Typically, ECFP4 or ECFP6, which involves two or three iterations, is widely used in the literature.[2–4] However, in this study, we employed a radius of 6, resulting in a more comprehensive fingerprint that captures a broader range of molecular substructures.

Duplicate substructures are identified and removed by comparing the sets of bonds they represent, ensuring uniqueness. The final ECFP fingerprint is compiled from all unique identifiers generated. For machine learning applications, these identifiers are often folded into a fixed-length bit vector through a modulo operation, ensuring a consistent size for computational tasks. In this study, a bit vector of 1024 bits was used.

Text S2 Machine Learning Models

Random Forest (RF)

RF uses multiple decision trees to improve predictive performance and robustness.[5] Each decision tree is based on a random subset of the training data and selected through bootstrapping. A random subset of features is used at each split to introduce diversity. This stochastic process reduces overfitting and improves generalization. The prediction of an RF is the aggregated output of all individual trees, which is the majority vote of the predicted classes for classification and the average of the predicted values for regression, respectively. The performance of an RF depends on the strength of the individual trees and the correlation between them. Lower correlation among trees leads to better performance by reducing variance. Out-of-bag (OOB) samples can be used with RF to estimate the generalization error and other performance metrics, providing a reliable measure of the model's predictive performance without requiring a separate validation set. This internal estimation mechanism ensures that the ensemble remains robust across various tasks.

eXtreme Gradient Boosting (XGBoost)

XGBoost is a gradient-boosted decision tree algorithm that leverages boosting to enhance predictive performance. It constructs a robust predictive model by integrating multiple weak learners, typically decision trees. The fundamental concept is to iteratively refine predictions by concentrating on samples that were previously misclassified or exhibited larger residuals. The model's prediction is expressed as the aggregate of predictions from all decision trees (Eq. 1):

$$\hat{y} = \sum_{k=1}^{K} f_k(x_i) \quad (1)$$

where f_k represents the k-th decision tree. To optimize the model, XGBoost minimizes a loss function that measures the difference between the predicted and actual values, while incorporating a regularization term to prevent overfitting (Eq. 2):

$$\mathcal{L} = \sum_i \ell(\hat{y}, y_i) + \sum_k \Omega(f_k), \text{ where } \Omega(f) = \gamma T + \frac{1}{2}\lambda \|\omega\|^2 \quad (2)$$

where $\ell(\hat{y}, y_i)$ is the total training loss between the prediction and training targets; $\Omega(f_k)$ penalizes the complexity of each tree, with T being the number of leaves, γ and λ as regularization parameters, and w as the weights of the leaves. To further enhance the model, XGBoost employs a second-order gradient boosting technique by using both the first derivative and the second derivative of the loss function. This approach allows for a more precise approximation of the loss function, leading to more efficient and effective tree construction. The algorithm also supports customizable loss functions, enabling it to be tailored to specific regression tasks, e.g., mean squared error (MSE) or mean absolute error (MAE). Refer to the studies by Chen & Guestrin, Shi & Wang [6,7] for details.

Multilayer Perceptron (MLP)

MLPs are a class of artificial neural networks that serve as powerful tools for pattern recognition and function approximation in various scientific domains. The MLP model consists of an input layer, one or more hidden layers, and an output layer, with each layer comprising a set of interconnected artificial neurons or nodes. The neurons are connected via weights, and the output of each neuron is determined by a nonlinear activation function applied to the weighted sum of its inputs. The fundamental operation within an MLP involves the forward propagation of input data through the network, where the input vector **x** is transformed into an output vector **y**. For a single neuron, this can be represented as (Eq. 3):[8]

$$y_i = f\left(\sum_{j=1}^{n} \omega_{ij}\, x_j + b_i\right) \quad (3)$$

where $f(\cdot)$ is the activation function, n is the number of inputs to neuron i, w_{ij} are the weights connecting the inputs x_j to the neuron, and b_i is the bias term. Training an MLP is performed by adjusting the weights and biases to minimize a cost function, such as the mean squared error (MSE), which quantifies the difference between the network's predictions and the true values.

Support Vector Regression (SVR)

SVR is a kernel-based supervised learning algorithm tailored for continuous prediction. It achieves robust performance by constructing an optimal regression hyperplane in a high-dimensional feature space so that as many data points as possible lie within a specified ε-insensitive tube, while minimizing the model complexity.[9] Training errors larger than ε are penalized through slack variables, and a regularization parameter C trades off model complexity against these errors. By means of the Gaussian radial basis function (RBF) or polynomial kernels, SVR can capture highly non-linear relationships in high-dimensional spaces, yet sparsity is retained because only the points lying outside or on the ε-tube (the support vectors) influence the final predictor.

Calculation of the MAE and RMSE

$$MAE = \frac{1}{N}\sum_{i=1}^{N}|y_i - \hat{y}_i|$$

$$RMSE = \sqrt{\frac{1}{N}\sum_{i=1}^{N}(y_i - \hat{y}_i)^2}$$

where y_i and $\hat{y}_i$ indicate the measured value and the predicted value, respectively.

Performance Comparison among the Four Models in This Study

The XGBoost model has the best performance, followed by the RF model. Both XGBoost and RF are decision tree-based models. Compared to RF, XGBoost builds trees

sequentially, each tree fitting the residuals left by the previous trees, thereby iteratively reducing prediction error and yielding higher R^2.[6] MLP, a feed-forward neural network model, can capture complex patterns in large data sets through its multilayered structure. However, the suboptimal performance of MLP in this study suggests that the limited size and complexity of our data set may not have been sufficient to fully leverage the capacity of MLP. Furthermore, SVR was less effective in capturing nonlinear relationships among multiple variables compared to XGBoost and RF.

Text S3 Permutation Feature Importance (PFI)

Permutation Feature Importance (PFI) is a model-agnostic method for assessing the importance of features in a predictive model. It measures the importance of a feature by evaluating the change in model performance subsequent to the random permutation of a feature's values.[10] Specifically, following the application of a trained model to generate predictions on a test data set, an initial reference score (rs), commonly represented by the coefficient of determination (R^2) in regression tasks, is established. For each feature, its values are randomly permuted within the data set to generate a corrupted version, upon which the model's performance is reassessed. The importance of the feature is subsequently determined by the discrepancy between the reference score and the score derived from the permuted data set (Eq. 4). A larger difference indicates a higher feature importance. To obtain a more stable estimate of feature importance, the permutation and score calculation steps are iterated multiple times (e.g., J times), with the mean difference adopted as the definitive importance metric (Eq. 5).

$$PFI_i = rs - s_i \tag{4}$$

$$PFI_i = rs - \frac{1}{J}\sum_{j=1}^{J} s_{i,j} \tag{5}$$

Text S4 Shapley Additive exPlanations (SHAP)

Shapley Additive exPlanations (SHAP) values quantify the contribution of each input feature to the prediction. Anchored in the Shapley values from cooperative game theory, which equitably apportions the collective payoffs among participants in a cooperative game, SHAP values allocate the model's prediction among its constituent features. As elucidated by Lundberg and Lee (2017),[11] the SHAP value for the *i*-th feature is computed as a weighted average of the discrepancies in model predictions when the feature is alternately included and excluded across all possible combinations of the remaining features (Eq. 6):

$$\emptyset_i = \sum_{S \subseteq F\{i\}} \frac{|S|!(|F| - |S| - 1)!}{|F|!} \left[f_{S\cup\{i\}}\left(x_{S\cup\{i\}}\right) - f_S(x_S) \right] \quad (6)$$

where F is the set of all input features; S signifies any subset of features in F excluding the *i*-th feature; $|S|$ and $|F|$ represent the number of elements in sets S and F, respectively; $f_{S\cup\{i\}}(x_{S\cup\{i\}})$ is the model's prediction when the *i*-th feature is incorporated; $f_S(x_S)$ denotes the model's prediction in the absence of the *i*-th feature; $x_{S\cup\{i\}}$ and x_S represent the input feature values with and without the *i*-th feature, respectively.

For linear models, SHAP values can be calculated directly. But for nonlinear models like tree-based models, specialized methodologies such as *TreeExplainer* are used to efficiently approximate these values. *TreeExplainer* takes advantage of the tree structure to consider all possible paths and prediction changes, providing a fast and accurate way to compute SHAP values and yielding profound insights into the model's predictive behavior.

Table S1 List of the 13 studies used for data set compilation

Title	Authors & Year	DOI/Link
Uptake and translocation of perfluoroalkyl acids (PFAA) in red chicory (*Cichorium intybus* L.) under various treatments with pre-contaminated soil and irrigation water	(Gredelj et al., 2020)	10.1016/j.scitotenv.2019.134766
Three potential sources of per- and polyfluoroalkyl substance exposure	(Lasee, 2020)	https://hdl.handle.net/2346/86735
Perfluoroalkyl acid distribution in various plant compartments of edible crops grown in biosolids-amended soils	(Blaine et al., 2014)	10.1021/es500016s
Uptake of perfluoroalkyl acids into edible crops via land applied biosolids: field and greenhouse studies	(Blaine et al., 2013)	10.1021/es403094q
The roles of protein and lipid in the accumulation and distribution of perfluorooctane sulfonate (PFOS) and perfluorooctanoate (PFOA) in plants grown in biosolids-amended soils	(Wen et al., 2016)	10.1016/j.envpol.2016.06.032
Uptake of perfluorinated alkyl acids by crops: results from a field study	(Felizeter et al., 2021)	10.1039/D1EM00166C
Field study on the uptake and translocation of perfluoroalkyl acids (PFAAs) by wheat (*Triticum aestivum* L.) grown in biosolids-amended soils	(Wen et al., 2014)	10.1016/j.envpol.2013.09.040
Behavior of N-ethyl perfluorooctane sulfonamido acetic acid (N-EtFOSAA) in biosolids amended soil-plant microcosms of seven plant species: Accumulation and degradation	(Wen et al., 2018)	10.1016/j.scitotenv.2018.06.073
Uptake of perfluorooctanoic acid, perfluorooctane sulfonate and perfluorooctane sulfonamide by carrot and lettuce from compost amended soil	(Bizkarguenaga et al., 2016)	10.1016/j.scitotenv.2016.07.010
Uptake and translocation of multiresidue industrial and household contaminants in radish grown under controlled conditions	(Abril et al., 2021)	10.1016/j.chemosphere.2020.128823
Commercial compost amendments inhibit the bioavailability and plant uptake of per- and polyfluoroalkyl substances in soil-porewater-lettuce systems	(Li et al., 2024)	10.1016/j.envint.2024.108615
Cultivar-dependent accumulation and translocation of perfluorooctanesulfonate among lettuce (*Lactuca sativa* L.) cultivars grown on perfluorooctanesulfonate-contaminated soil	(Yu et al., 2018)	10.1021/acs.jafc.8b04548
Genotypic variation and mechanism in uptake and translocation of perfluorooctanoic acid (PFOA) in lettuce (*Lactuca sativa* L.) cultivars grown in PFOA-polluted soils	(Xiang et al., 2018)	10.1016/j.scitotenv.2018.04.354

Table S2 All input variables for model prediction

	**Categorical variable**	**Types**	**Numerical variable**	**Range**
Plant	Species	11	Growth time (days)	32~258
	Categories	5		
	Tissues	11	-	
	Casparian strip	-		
PFAS	Compounds	21	Molar mass (g/mol)	180.03~714.11
	ECFP	1024	Carbon chain length	2~13
	Functional group	4	-	
Soil	-		pH	4~8.75
			SOM (%)	0.086~10.93
			Sand (%)	10.7~100
			Silt (%)	0~79.4
			Clay (%)	0~26
			Cation exchange capacity (cmol/kg)	0.02~130
			Initial concentration in soil (ng/g)	0.025~10581
Cultivation	Cultivation mode	2	Additional input (μg)	0~2352

Table S3 The category, species and tissue of plant collected in the data set

**Category (Samples)**	**Species**	**Tissues**
Leafy (721)	Red chicory (*Cichorium intybus* L.)<br>Lettuce (*Lactuca sativa* L.)<br>Alfalfa (*Medicago sativa* L.)	Root; shoot; leaves; stem
Root (298)	Radish (*Raphanus sativus* L.)<br>Carrot (*Daucus carota* subsp. sativus)<br>Celery (*Apium graveolens* L.)	Root; shoot; leaves; stem
Fruit (120)	Tomato (*Solanum lycopersicum*)	Root; shoot; leaves; stem; fruit
Cereal (552)	Wheat (*Triticum aestivum* L.)<br>Maize (*Zea mays* L.)	Root; shoot; leaves; stem; grain; husk; hull-leaves; cob
Legume (310)	Sugar snap pea (*Pisum sativum*)<br>Pea (*Pisum sativum*)	Root; shoot; leaves; stem; pea; pod

Table S4 Names and structures of PFAS collected in the data set

**Compound**	**SMILES**	**Chemical formula**	**Functional group**	**p*K*a**
PFTeA	C(=O)(C(C(C(C(C(C(C(C(C(C(C(C(C(F)(F)F)(F)F)(F)F)(F)F)(F)F)(F)F)(F)F)(F)F)(F)F)(F)F)(F)F)(F)F)(F)F)O	$C_{13}F_{27}COOH$	-COOH	0.50[12]
PFTrDA	C(=O)(C(C(C(C(C(C(C(C(C(C(C(C(F)(F)F)(F)F)(F)F)(F)F)(F)F)(F)F)(F)F)(F)F)(F)F)(F)F)(F)F)(F)F)O	$C_{12}F_{25}COOH$	-COOH	0.50[12]
PFDoA	C(=O)(C(C(C(C(C(C(C(C(C(C(C(F)(F)F)(F)F)(F)F)(F)F)(F)F)(F)F)(F)F)(F)F)(F)F)(F)F)(F)F)O	$C_{11}F_{23}COOH$	-COOH	-0.17[13]
PFUnA	C(=O)(C(C(C(C(C(C(C(C(C(C(F)(F)F)(F)F)(F)F)(F)F)(F)F)(F)F)(F)F)(F)F)(F)F)(F)F)O	$C_{10}F_{21}COOH$	-COOH	-0.17[13]
PFDA	C(=O)(C(C(C(C(C(C(C(C(C(F)(F)F)(F)F)(F)F)(F)F)(F)F)(F)F)(F)F)(F)F)(F)F)O	$C_9F_{19}COOH$	-COOH	-0.17[13]
PFNA	C(=O)(C(C(C(C(C(C(C(C(F)(F)F)(F)F)(F)F)(F)F)(F)F)(F)F)(F)F)(F)F)O	$C_8F_{17}COOH$	-COOH	-0.21[13]
PFOA	C(=O)(C(C(C(C(C(C(C(F)(F)F)(F)F)(F)F)(F)F)(F)F)(F)F)(F)F)O	$C_7F_{15}COOH$	-COOH	-0.50[14]
PFHpA	C(=O)(C(C(C(C(C(C(F)(F)F)(F)F)(F)F)(F)F)(F)F)(F)F)O	$C_6F_{13}COOH$	-COOH	-0.15[13]
PFHxA	C(=O)(C(C(C(C(C(F)(F)F)(F)F)(F)F)(F)F)(F)F)O	$C_5F_{11}COOH$	-COOH	-0.16[15]
PFPeA	C(=O)(C(C(C(C(F)(F)F)(F)F)(F)F)(F)F)O	C_4F_9COOH	-COOH	-0.20*
PFBA	C(=O)(C(C(C(F)(F)F)(F)F)(F)F)O	$C3F_7COOH$	-COOH	0.08[13]
GenX	C(=O)(C(C(F)(F)F)(OC(C(C(F)(F)F)(F)F)(F)F)F)O	$C_3F_7OCF(CF_3)COOH$	-COOH	-0.10[16]
PFMOAA	C(=O)(C(OC(F)(F)F)(F)F)O	CF_3OCF_2COOH	-COOH	0.10[12]
C8/C8 PFPiA	C(C(C(C(C(F)(F)P(=O)(C(C(C(C(C(C(C(C(F)(F)F)(F)F)(F)F)(F)F)(F)F)(F)F)(F)F)(F)F)O)(F)F)(F)F)(F)F)(C(C(C(F)(F)F)(F)F)(F)F)(F)F	$C_8F_{17}PO_2HC_8F_{17}$	$-PO_2H$	-1.40*
PFOSA	C(C(C(C(C(F)(F)S(=O)(=O)N)(F)F)(F)F)(F)F)(C(C(C(F)(F)F)(F)F)(F)F)(F)F	$C_8F_{17}SO_2NH_2$	$-SO_2NH_2$	6.24[13]
PFOS	C(C(C(C(C(F)(F)S(=O)(=O)O)(F)F)(F)F)(F)F)(C(C(C(F)(F)F)(F)F)(F)F)(F)F	$C_8F_{17}SO_3H$	$-SO_3H$	0.14[13]
6:2 Cl-PFESA	C(C(C(C(F)(F)Cl)(F)F)(F)F)(C(C(OC(C(F)(F)S(=O)(=O)O)(F)F)(F)F)(F)F)(F)F	$C_6ClF_{12}OC_2F_4SO_3H$	$-SO_3H$	0.10*
C6/C6 PFPiA	C(C(C(C(F)(F)P(=O)(C(C(C(C(C(C(F)(F)F)(F)F)(F)F)(F)F)(F)F)(F)F)O)(F)F)(F)F)(C(C(F)(F)F)(F)F)(F)F	$C_6F_{13}PO_2HC6F_{13}$	$-PO_2H$	-1.4[17]
PFHxS	C(C(C(C(F)(F)S(=O)(=O)O)(F)F)(F)F)(C(C(F)(F)F)(F)F)(F)F	$C_6F_{13}SO_3H$	$-SO_3H$	0.14[13]
PFBS	C(C(C(F)(F)S(=O)(=O)O)(F)F)(C(F)(F)F)(F)F	$C_4F_9SO_3H$	$-SO_3H$	0.14[13]
8:2 FTS	C(CS(=O)(=O)O)C(C(C(C(C(C(C(C(F)(F)F)(F)F)(F)F)(F)F)(F)F)(F)F)(F)F)(F)F	$C_8F_{17}C_2H_4SO_3H$	$-SO_3H$	0.40*
6:2 FTS	C(CS(=O)(=O)O)C(C(C(C(C(C(F)(F)F)(F)F)(F)F)(F)F)(F)F)(F)F	$C_6F13C_2H_4SO_3H$	$-SO_3H$	0.40[13]

N-EtFOSAA	CCN(CC(=O)O)S(=O)(=O)C(C(C(C(C(C(C(C(F)(F)F)(F)F)(F)F)(F)F)(F)F)(F)F)(F)F)(F)F	$C_8F_{17}SO_2N(C_2H_5)CH_2COOH$	-COOH/-SO_2	3.90[18]
4:2 FTS	C(CS(=O)(=O)O)C(C(C(C(F)(F)F)(F)F)(F)F)(F)F	$C_4F_9C_2H_4SO_3H$	-SO_3H	0.40*

Note: [13] pKa values were obtained using the SPARC; *, based on other similar chemical structures.

Table S5 Hyperparameters for the machine learning procedure

Model	Hyperparameters	Log BCF	Log C
XGBoost	n_estimators	120	120
	learning_rate	0.1	0.1
	max_depth	9	7
	min_child_weight	1	3
	gamma	0	0
	Subsample	0.7	0.8
RF	n_estimators	100	110
	max_depth	19	21
	min_samples_leaf	1	1
	min_samples_split	2	2
	bootstrap	True	True
MLP	hidden_layer_sizes	(84)	(66)
	learning_rate_init	0.01	0.03
	activation	relu	relu
	alpha	0.07	0.01
	max_iter	200	1000
SVR	C	4	38
	gamma	0.01	0.26
	epsilon	0.27	0.28
	Kernel	rbf	rbf

Table S6 Performance metrics of four machine learning models (XGBoost, RF, MLP, SVR) on Log BCF and Log C predictions

**Model**	**Subset**	**Log BCF**			**Log C**		
		**RMSE**	**MAE**	**R^2**	**RMSE**	**MAE**	**R^2**
XGBoost	Train	0.105	0.0678	0.996	0.160	0.115	0.990
	Validation	0.386	0.242	0.938	0.450	0.284	0.919
	Test	0.383	0.239	0.951	0.386	0.253	0.943
RF	Train	0.180	0.119	0.987	0.178	0.116	0.987
	Validation	0.419	0.258	0.927	0.478	0.300	0.909
	Test	0.386	0.247	0.951	0.442	0.297	0.925
MLP	Train	0.824	0.599	0.737	0.866	0.668	0.700
	Validation	0.797	0.582	0.737	0.871	0.658	0.697
	Test	0.847	0.632	0.762	0.927	0.715	0.672
SVR	Train	0.543	0.341	0.886	0.501	0.320	0.899
	Validation	0.579	0.392	0.861	0.853	0.580	0.710
	Test	0.714	0.468	0.830	0.855	0.597	0.722

RMSE, root mean square error; MAE, mean absolute error; R^2, coefficient of determination.

Table S7 Performance of the Log C and Log BCF models across soil PFAS concentration ranges in the test set

Soil concentration group (ng/g)		<10	10 – 100	100 – 1000	≥1000	Overall test set
Minimum (ng/g)		0.025	10.2	100.3	1001	0.025
Maximum (ng/g)		9.04	98.8	990.3	10581	10581
n		36	48	51	66	201
Log BCF	Observed target variance	2.80	1.86	2.18	2.66	3.02
	R^2	0.88	0.95	0.95	0.96	0.95
	RMSE	0.56	0.31	0.34	0.34	0.38
	MAE	0.32	0.21	0.22	0.23	0.24
Log C	Observed target variance	2.51	1.73	2.06	2.56	2.64
	R^2	0.88	0.90	0.94	0.97	0.94
	RMSE	0.54	0.41	0.34	0.29	0.39
	MAE	0.33	0.26	0.25	0.21	0.25

Table S8 Feature Importance Scores from PFI and SHAP

Log BCF				Log C			
Feature	PFI	**Feature**	**SHAP**	**Feature**	**PFI**	**Feature**	**SHAP**
pH	1.63	pH	0.82	C (Initial in soil)	1.80	C (Initial in soil)	0.62
Carbon chain length	1.00	Carbon chain length	0.35	Growth time	0.60	Growth time	0.44
Plant tissues_Root	0.64	Plant tissues_Root	0.25	Carbon chain length	0.48	Carbon chain length	0.27
Plant species_Red chicory	0.41	Plant species_Red chicory	0.21	Plant tissues_Root	0.48	Plant tissues_Root	0.21
CEC	0.37	Plant tissues_Leaves	0.14	CEC	0.19	CEC	0.14
Plant tissues_Leaves	0.23	CEC	0.12	Plant tissues_Leaves	0.16	Plant tissues_Leaves	0.11
C (Initial in soil)	0.21	Growth time	0.11	pH	0.13	Soil pH	0.10
Growth time	0.09	C (Initial in soil)	0.09	Sand	0.07	ECFP_898	0.09
Plant tissues_Grain	0.05	Plant tissues_Grain	0.05	Plant tissues_Peas	0.07	Sand	0.07
Plant species_Lettuce	0.05	Plant tissues_Peas	0.05	ECFP_898	0.07	ECFP_846	0.07
Plant tissues_Peas	0.04	Plant tissues_Shoot	0.04	Plant tissues_Grain	0.05	Plant tissues_Peas	0.05
Plant tissues_Stem	0.04	SOM	0.04	Plant tissues_Shoot	0.04	Plant tissues_Shoot	0.05
SOM	0.04	Plant species_Lettuce	0.03	SOM	0.04	SOM	0.05
ECFP_969	0.03	Plant tissues_Stem	0.03	Additional input	0.03	Plant tissues_Grain	0.04
Additional input	0.03	Plant species_Pea	0.02	ECFP_846	0.03	Additional input	0.04
ECFP_863	0.02	Casparian strip	0.02	ECFP_808	0.02	ECFP_973	0.03
ECFP_808	0.02	Plant tissues_Fruit	0.02	Plant tissues_Stem	0.02	Cultivation condition	0.02
Plant tissues_Fruit	0.02	ECFP_969	0.02	Plant species_Lettuce	0.02	Plant species_Lettuce	0.02
Plant tissues_Shoot	0.02	Additional input	0.02	Plant tissues_Pods	0.02	Plant tissues_Pods	0.02
Casparian strip	0.01	Functional groups_Carboxylate	0.02	Cultivation condition	0.01	ECFP_57	0.02

Table S9 The important substructures affecting BCF and concentration of PFAS

ECFP	Substructure
ECFP-969	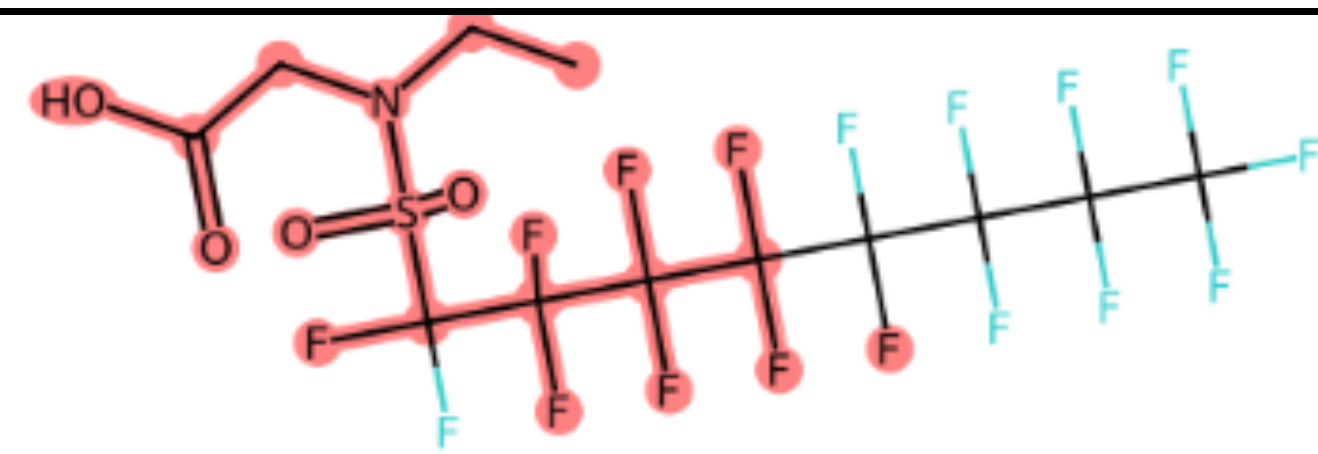
ECFP-898	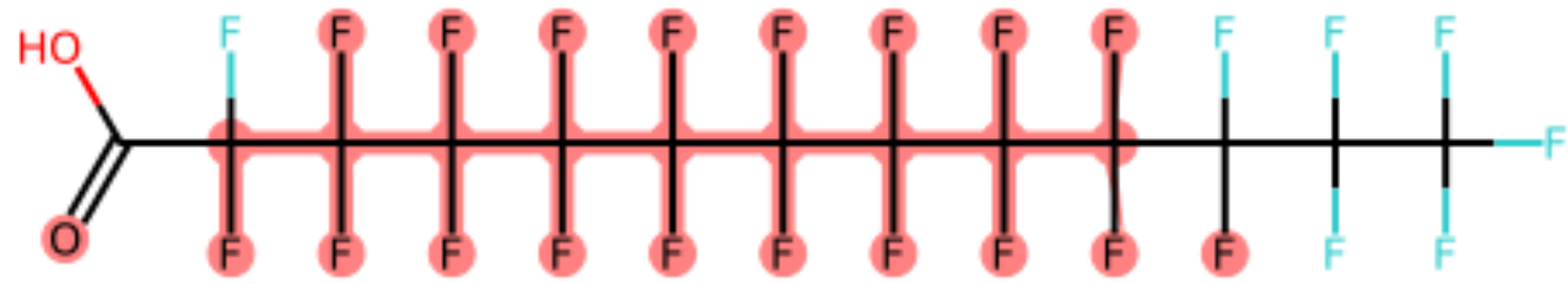
ECFP-846	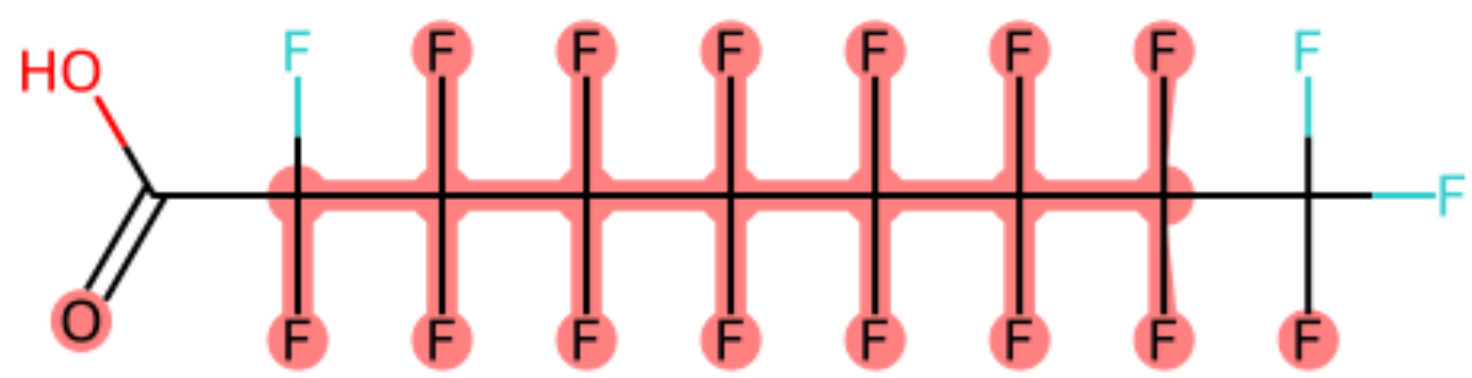
ECFP-973	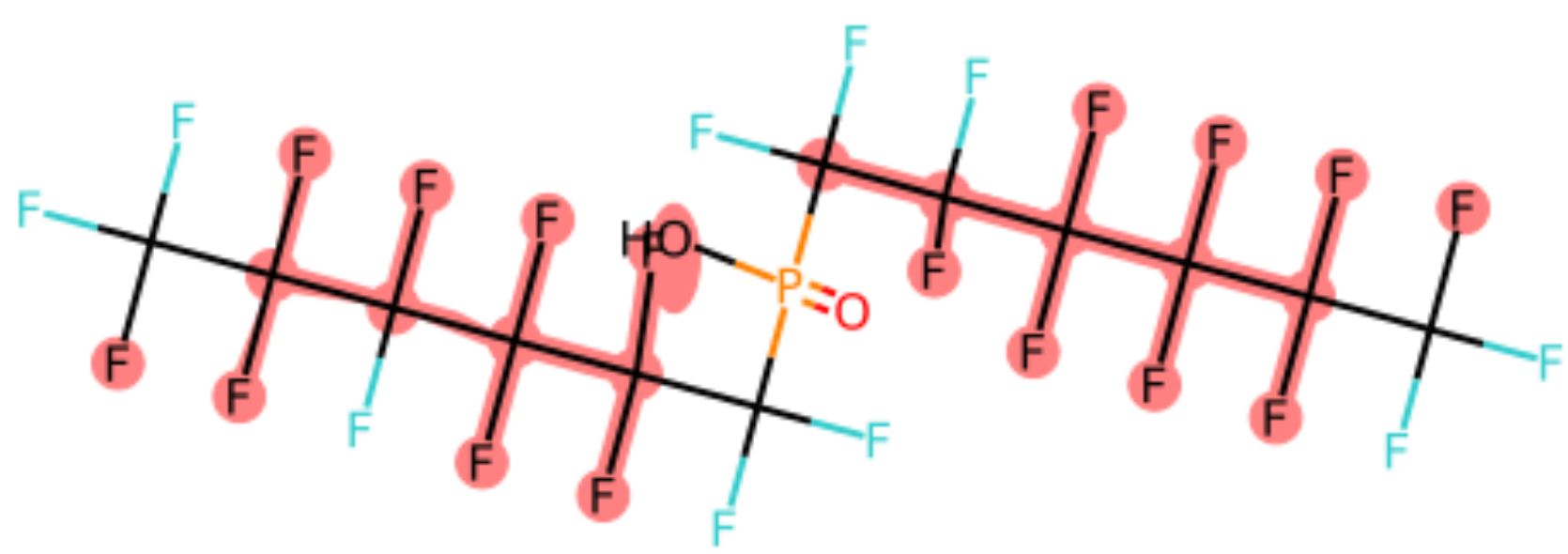
ECFP-57	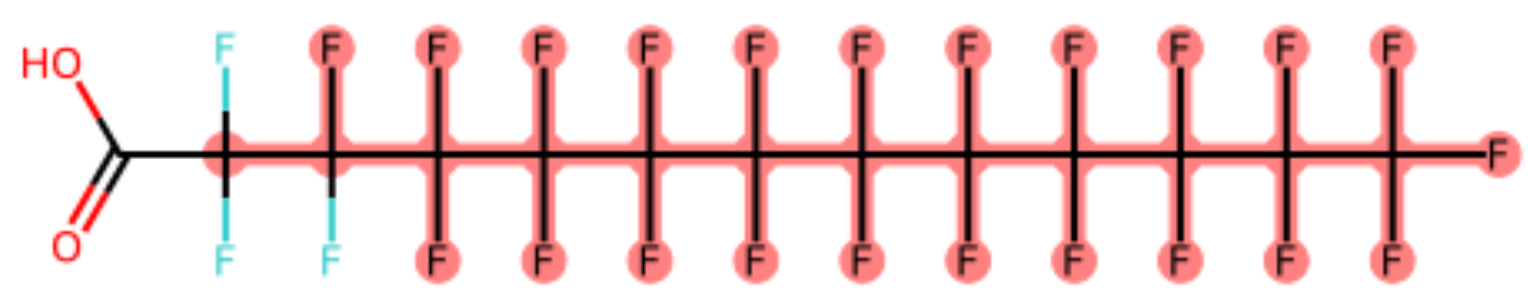

Chemical selected randomly only to show key substructures.

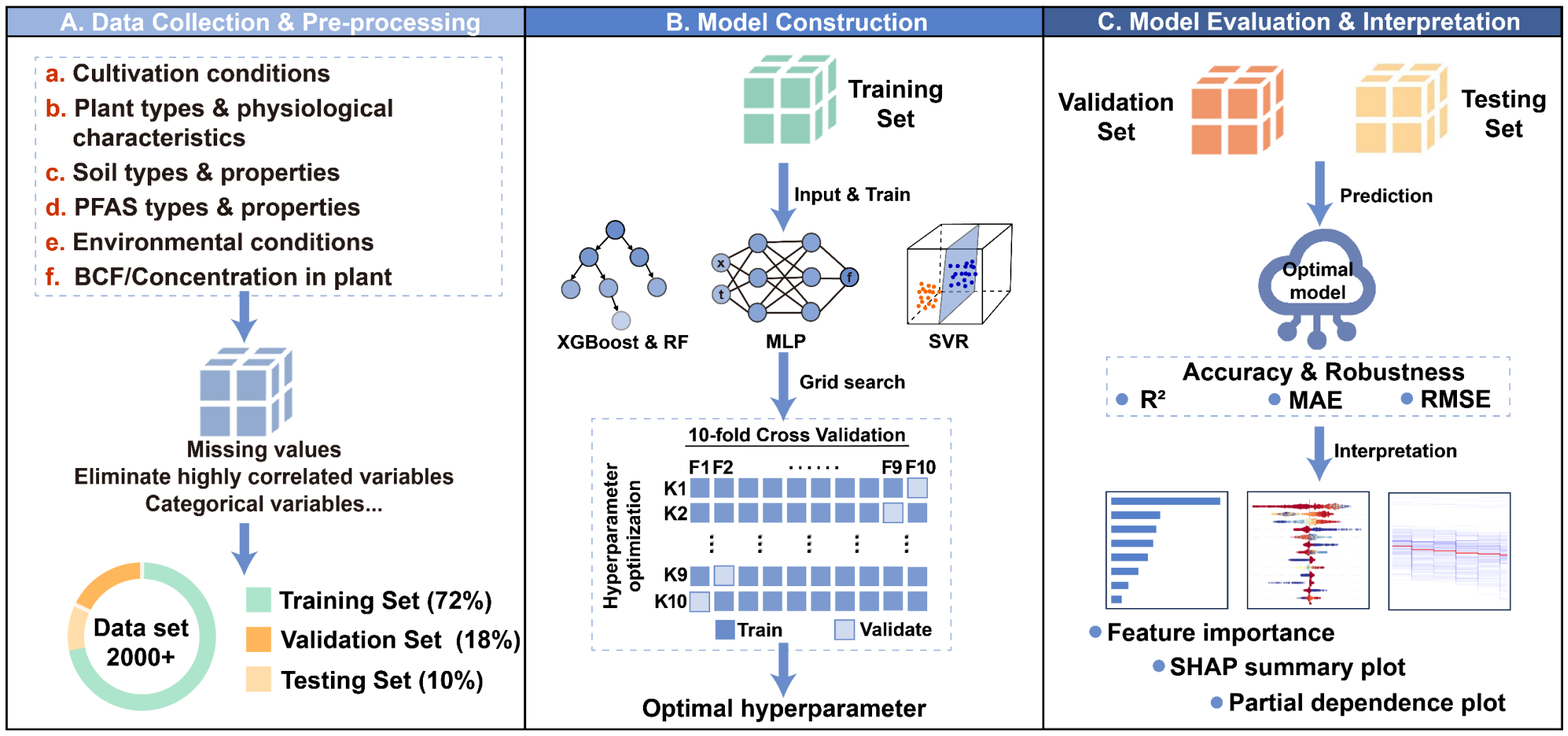

Figure S1. Referential logical framework for predicting plant uptake of PFAS based on machine learning model

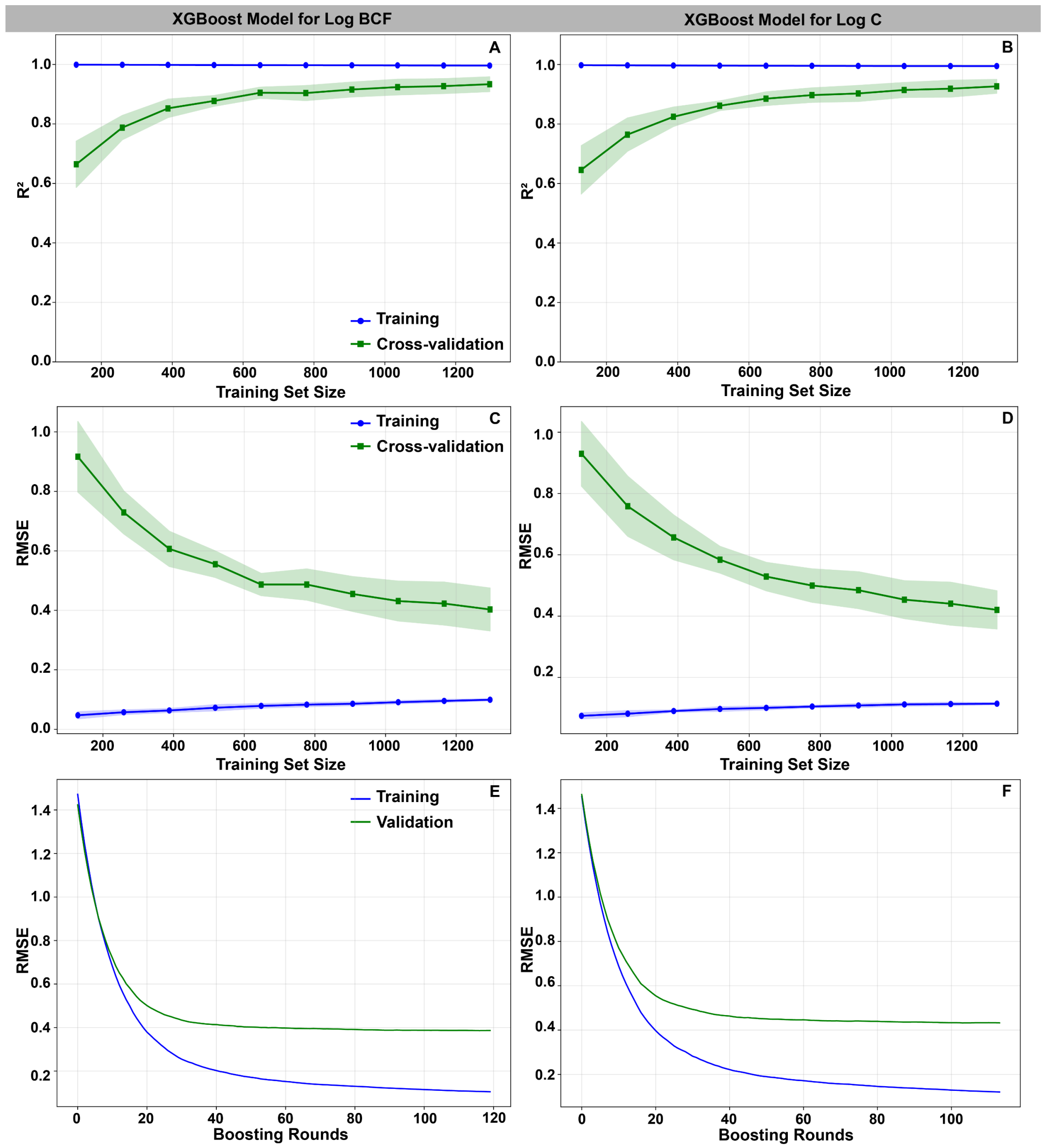

Figure S2. Learning curves (A-D) and early stopping (E-F) analysis for the XGBoost models. R^2, coefficient of determination; RMSE, root mean square error

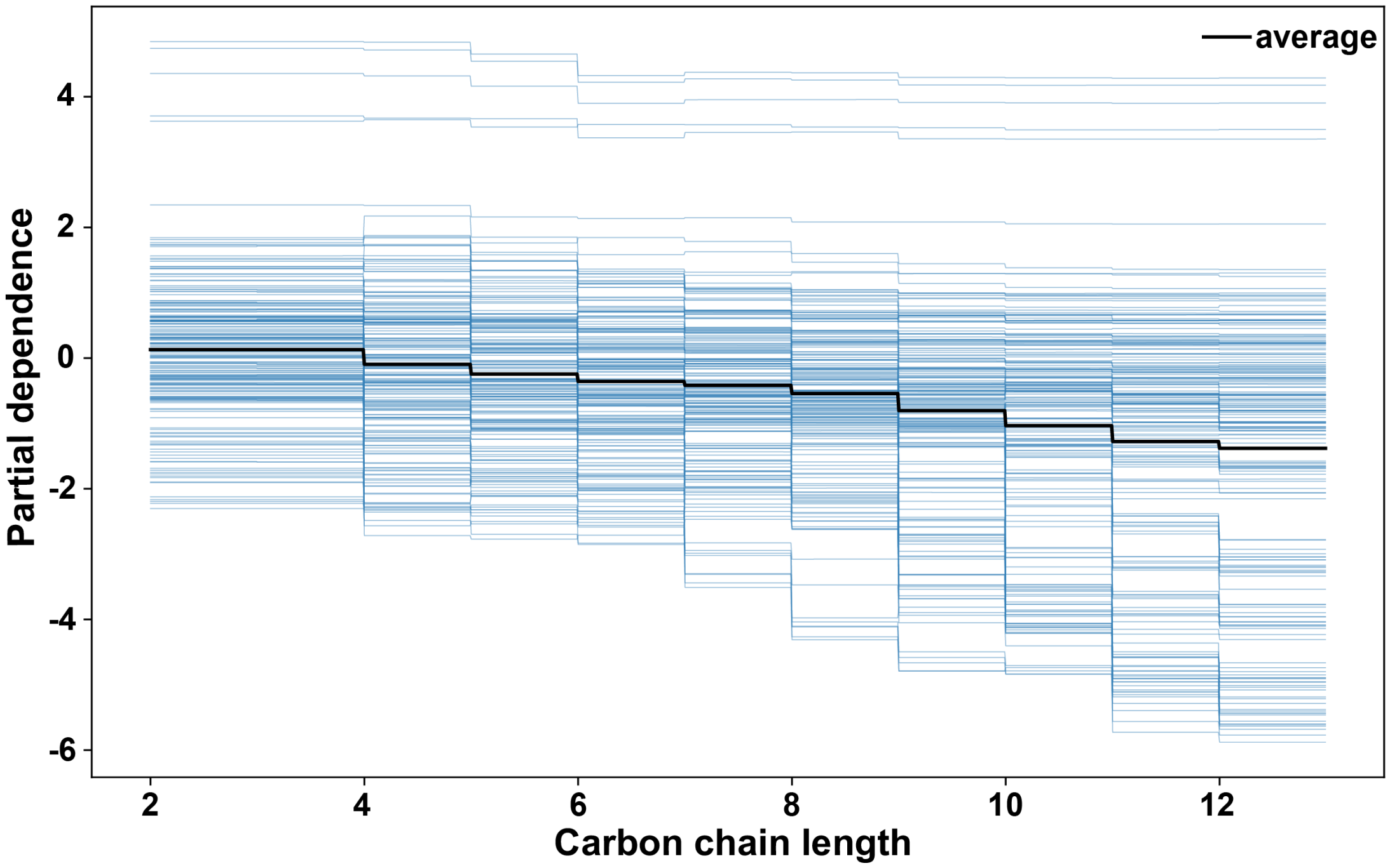

Figure S3. Individual conditional expectation (ICE) and partial dependence plot (PDP) of PFAS carbon chain length on Log BCF

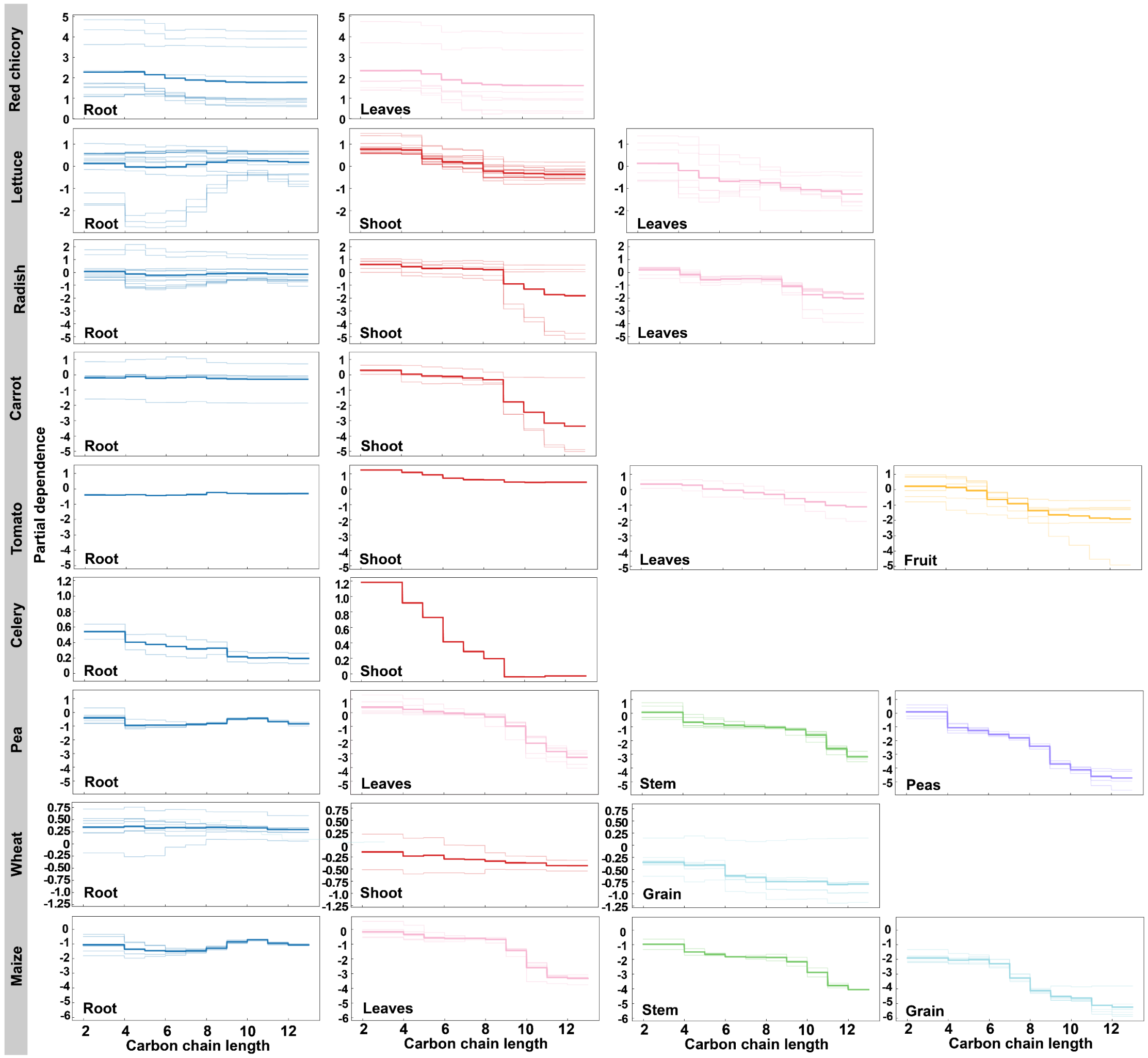

Figure S4. ICE and PDP of PFAS carbon chain length on Log BCF in various plant tissues; bold lines indicate the average

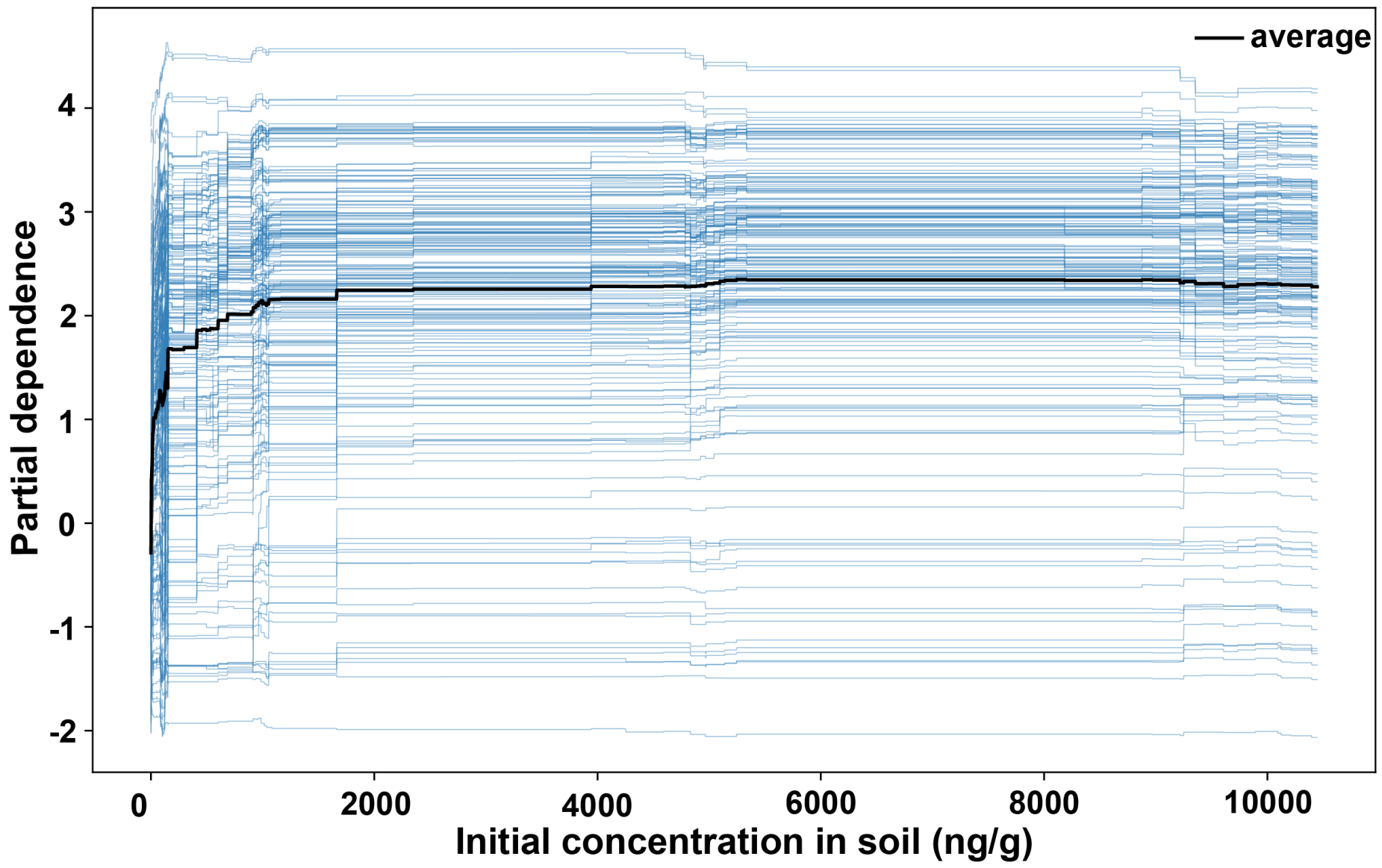

Figure S5. ICE and PDP of initial concentration in soil on Log C

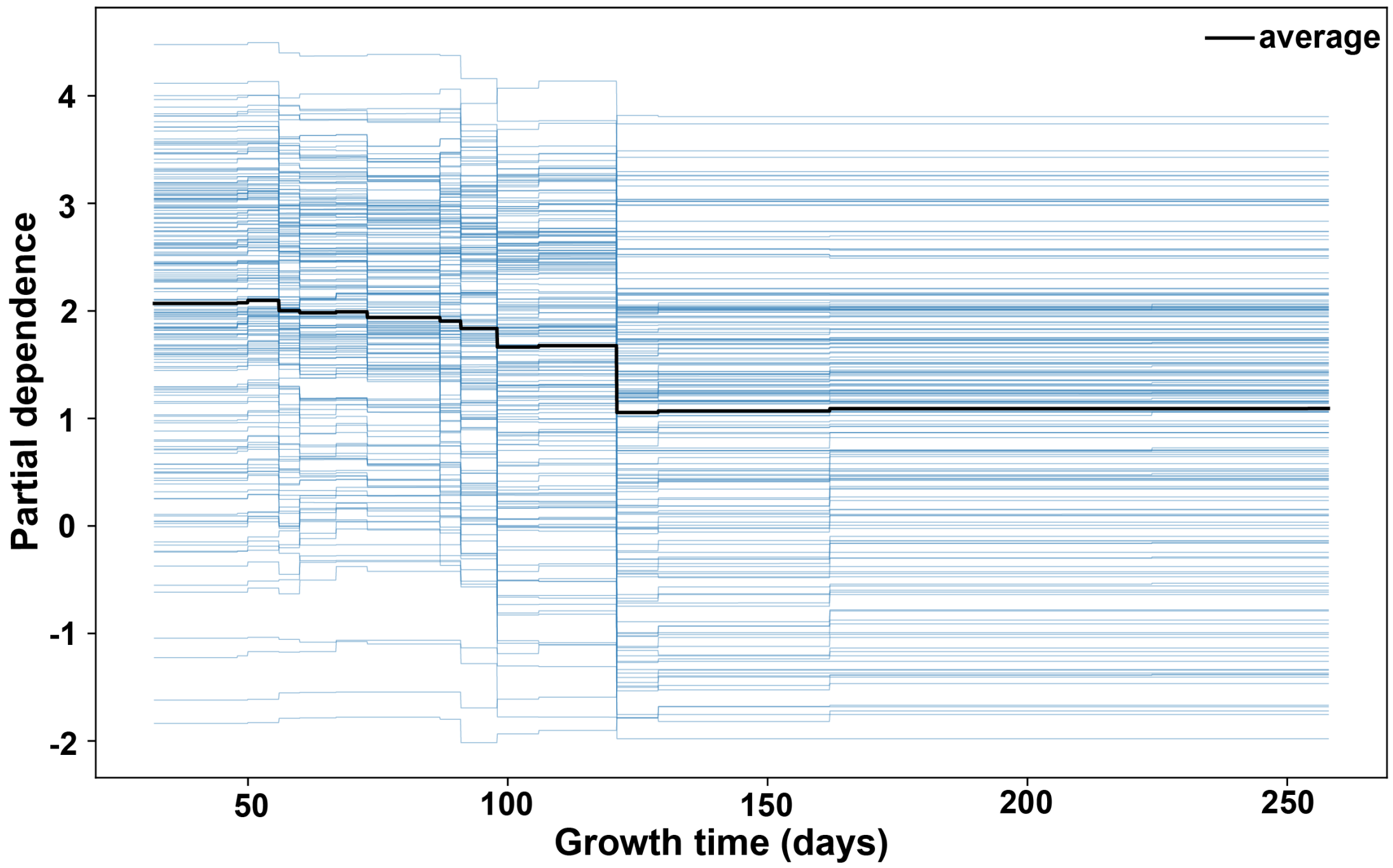

Figure S6. ICE and PDP of plant growth time on Log C

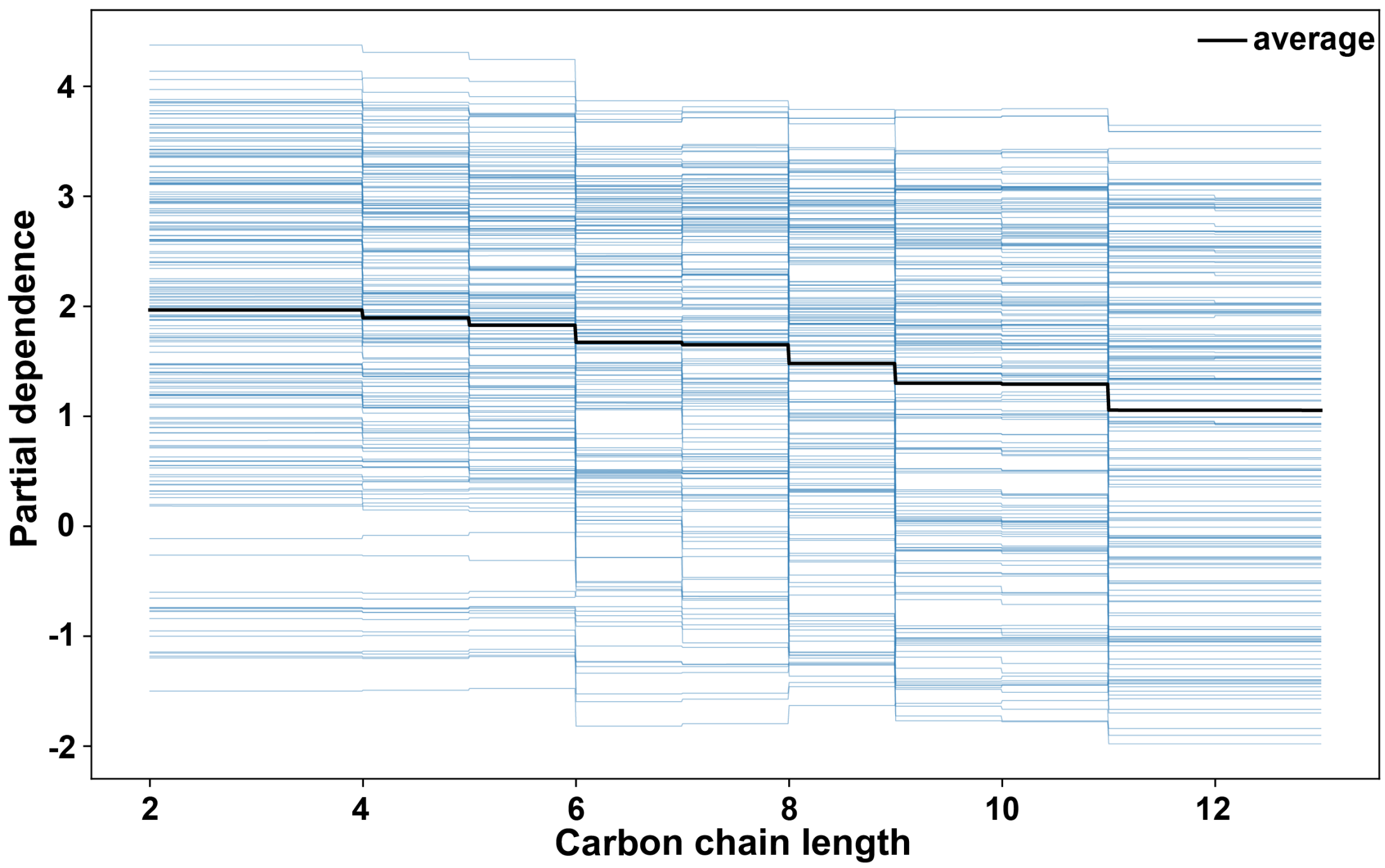

Figure S7. ICE and PDP of PFAS carbon chain length on Log C

References

(1) Rogers, D.; Hahn, M. Extended-Connectivity Fingerprints. *J. Chem. Inf. Model.* **2010**, *50* (5), 742–754. https://doi.org/10.1021/ci100050t.

(2) Gao, F.; Shen, Y.; Sallach, J. B.; Li, H.; Liu, C.; Li, Y. Direct Prediction of Bioaccumulation of Organic Contaminants in Plant Roots from Soils with Machine Learning Models Based on Molecular Structures. *Environ. Sci. Technol.* **2021**, *55* (24), 16358–16368. https://doi.org/10.1021/acs.est.1c02376.

(3) Song, C.; Gu, Q.; Zhang, D.; Zhou, D.; Cui, X. Prediction of PFAS Bioaccumulation in Different Plant Tissues with Machine Learning Models Based on Molecular Fingerprints. *Science of The Total Environment* **2024**, *950*, 175091. https://doi.org/10.1016/j.scitotenv.2024.175091.

(4) Myint, K.-Z.; Wang, L.; Tong, Q.; Xie, X.-Q. Molecular Fingerprint-Based Artificial Neural Networks QSAR for Ligand Biological Activity Predictions. *Mol. Pharmaceutics* **2012**, *9* (10), 2912–2923. https://doi.org/10.1021/mp300237z.

(5) Breiman, L. Random Forests. *Machine Learning* **2001**, *45* (1), 5–32. https://doi.org/10.1023/A:1010933404324.

(6) Chen, T.; Guestrin, C. XGBoost: A Scalable Tree Boosting System. In *Proceedings of the 22nd ACM SIGKDD International Conference on Knowledge Discovery and Data Mining*; KDD '16; Association for Computing Machinery: New York, NY, USA, **2016**; pp 785–794. https://doi.org/10.1145/2939672.2939785.

(7) Shi, C.; Wang, Y. Development of Subsurface Geological Cross-Section from Limited Site-Specific Boreholes and Prior Geological Knowledge Using Iterative Convolution XGBoost. *Journal of Geotechnical and Geoenvironmental Engineering* **2021**, *147* (9), 04021082. https://doi.org/10.1061/(ASCE)GT.1943-5606.0002583.

(8) Popescu, M.-C.; Balas, V. E.; Perescu-Popescu, L.; Mastorakis, N. Multilayer Perceptron and Neural Networks. *WSEAS Trans. Circuits Syst.* **2009**, *8* (7), 579–588.

(9) Smola, A. J.; Schölkopf, B. A Tutorial on Support Vector Regression. *Statistics and Computing* **2004**, *14* (3), 199–222. https://doi.org/10.1023/B:STCO.0000035301.49549.88.

(10) Kaneko, H. Cross-Validated Permutation Feature Importance Considering Correlation between Features. *Analytical Science Advances* **2022**, *3* (9–10), 278–287. https://doi.org/10.1002/ansa.202200018.

(11) Lundberg, S. M.; Lee, S.-I. A Unified Approach to Interpreting Model Predictions. In *Advances in Neural Information Processing Systems*; Curran Associates, Inc., **2017**; Vol. 30.

(12) *ChemicalBook*. https://www.chemicalbook.com/ (accessed 2026-02-01).

(13) Hilal, S. H.; Karickhoff, S. W.; Carreira, L. A. A Rigorous Test for SPARC's Chemical Reactivity Models: Estimation of More Than 4300 Ionization pKas. *Quantitative Structure-Activity Relationships* **1995**, *14* (4), 348–355. https://doi.org/10.1002/qsar.19950140405.

(14) Goss, K.-U. The pKa Values of PFOA and Other Highly Fluorinated Carboxylic Acids. *Environ. Sci. Technol.* **2008**, *42* (2), 456–458. https://doi.org/10.1021/es702192c.

(15) Zhao, Y. G.; Wan, H. T.; Wong, M. H.; Wong, C. K. C. Partitioning Behavior of Perfluorinated Compounds between Sediment and Biota in the Pearl River Delta of South China. *Marine Pollution Bulletin* **2014**, *83* (1), 148–154. https://doi.org/10.1016/j.marpolbul.2014.03.060.

(16) Xiao, F. Emerging Poly- and Perfluoroalkyl Substances in the Aquatic Environment: A Review of Current Literature. *Water Research* **2017**, *124*, 482–495. https://doi.org/10.1016/j.watres.2017.07.024.
(17) Gao, Z.; Zhou, J.; Xue, M.; Liu, S.; Guo, J.; Zhang, Y.; Cao, C.; Wang, T.; Zhu, L. Theoretical and Experimental Insights into the Mechanisms of C6/C6 PFPiA Degradation by Dielectric Barrier Discharge Plasma. *Journal of Hazardous Materials* **2022**, *424*, 127522. https://doi.org/10.1016/j.jhazmat.2021.127522.
(18) Rayne, S. Comment on "Indirect Photolysis of Perfluorochemicals: Hydroxyl Radical-Initiated Oxidation of N-Ethyl Perfluorooctane Sulfonamido Acetate (N-EtFOSAA) and Other Perfluoroalkanesulfonamides." *Environ. Sci. Technol.* **2009**, *43* (20), 7995–7996. https://doi.org/10.1021/es9022464.
